# Red blood cells: a potential delivery system

**DOI:** 10.1186/s12951-023-02060-5

**Published:** 2023-08-22

**Authors:** Mengran Chen, Yamei Leng, Chuan He, Xuefeng Li, Lei Zhao, Ying Qu, Yu Wu

**Affiliations:** 1 Department of Hematology, West China Hospital, Sichuan University/West China School of Nursing, Sichuan University, Chengdu, 610041 Sichuan People’s Republic of China; 2 Guang’an People’s Hospital, Guang’an, 638001 Sichuan People’s Republic of China

**Keywords:** Red blood cells, Delivery system, Therapeutic strategies, Bioimaging

## Abstract

Red blood cells (RBCs) are the most abundant cells in the body, possessing unique biological and physical properties. RBCs have demonstrated outstanding potential as delivery vehicles due to their low immunogenicity, long-circulating cycle, and immune characteristics, exhibiting delivery abilities. There have been several developments in understanding the delivery system of RBCs and their derivatives, and they have been applied in various aspects of biomedicine. This article compared the various physiological and physical characteristics of RBCs, analyzed their potential advantages in delivery systems, and summarized their existing practices in biomedicine.

**Graphical Abstract:**

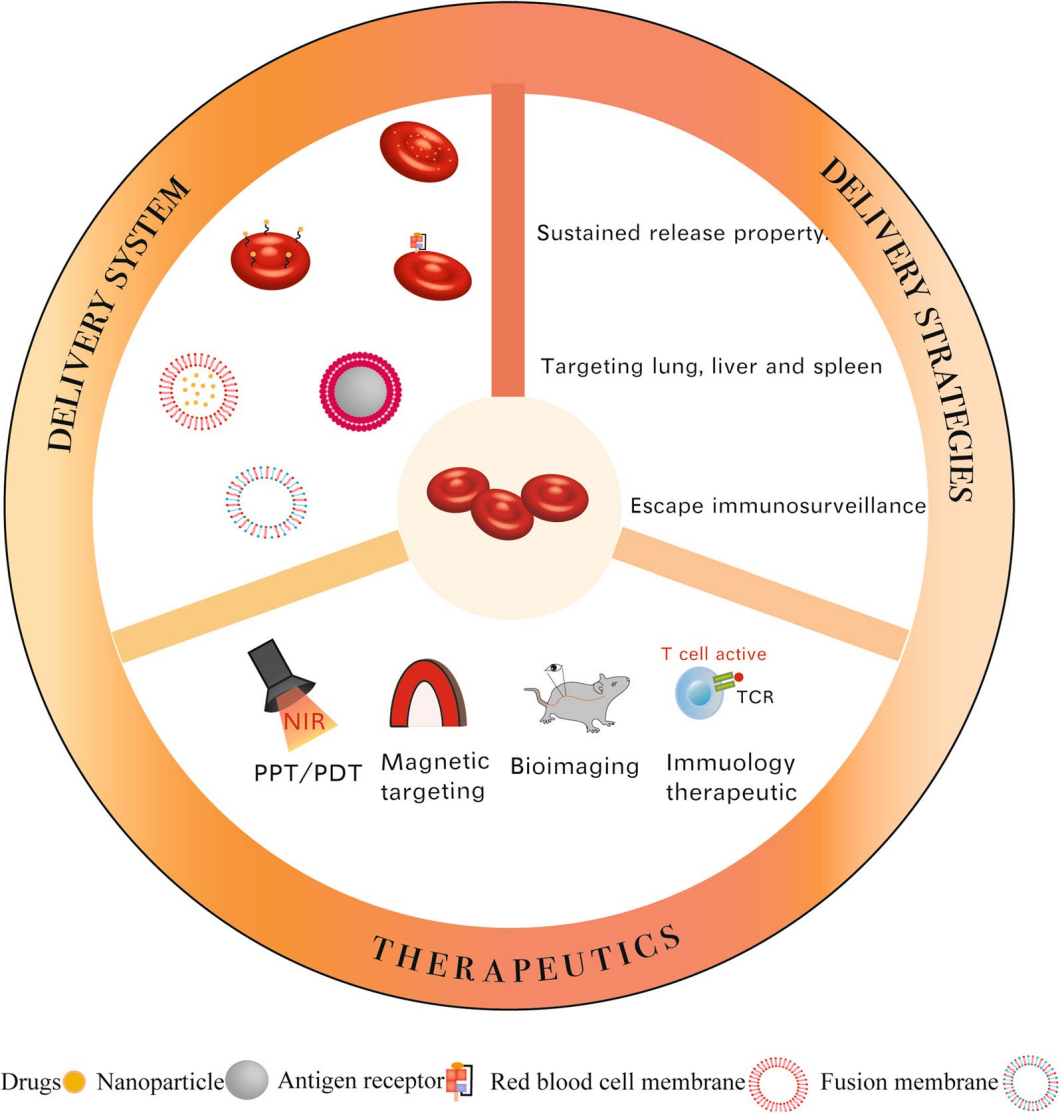

## Background

The development of drug delivery systems has become increasingly sophisticated in recent decades. Nanocarriers with rational designs could enhance the application of various diagnostic technologies and therapeutic strategies which are limited by low solubility, rapid elimination, severe side effects, poor biocompatibility, limited biodegradability, or expensive costs [1]. Currently, delivery materials, such as polymers [2], liposomes [3], metals [4], and molecules [5] have been utilized. Some materials have achieved good results in phase III trials and clinical practice [3, 4]. But researchers are still exploring new, more effective and convenient delivery systems. Biological drug delivery systems (bDDSs) are a hot topic in delivery systems [6] and are based on natural cells and their derivatives, with good biocompatibility and biofunctionality [7]. It seems that combining the physiological and physical characteristics of cells with delivery can improve delivery efficiency and expand application fields. The common materials used for bDDSs include erythrocytes, platelets, neutrophils, and various cell membranes or cell-derived vesicles.

Erythrocytes or red blood cells (RBCs) are the most abundant cells in the blood. By utilizing the RBCs’ inherent physical and chemical characteristics, the delivery system based on RBCs demonstrated good delivery efficiency and mimicked some natural mechanisms. As early as the 1970s, scientists have used RBC ghosts as carriers for delivery in vitro [8]. With bDDSs becoming increasingly mature, RBC drug delivery systems have been used in medical treatment [9, 10], immune therapy [6, 11], bioimaging [12] and many other biomedical fields.

The strategies for using RBCs as drug delivery carriers have been developed in many forms over the years. This article summarized the application of RBCs and their derivatives in biomedicine and attempted to explain the effects of different methods of RBC treatment on the delivery of drugs.

## Physical and physiological characteristics of RBCs

RBCs are functional cells in the human body, and their growth, development, and characteristics are like other cells. However, they have some unique manifestations. In order to exert the role of transporting oxygen, RBCs have formed some unique morphological and physiological characteristics.

### Structure and morphology of RBCs

RBCs are biconcave disk shape cells with special physical features. The biconcave disk shape increases RBC surface area and its deformability [13]. Their surface area of 140 µm^2^ exhibits an excess surface area of 40% compared with a sphere of the same volume. The human RBC diameter was approximately 8 μm [14], with a volume of 90 fL. However, they can pass through 1/16 times the size of endothelial slits in the red pulp of the spleen [15].

**Fig. 1:**
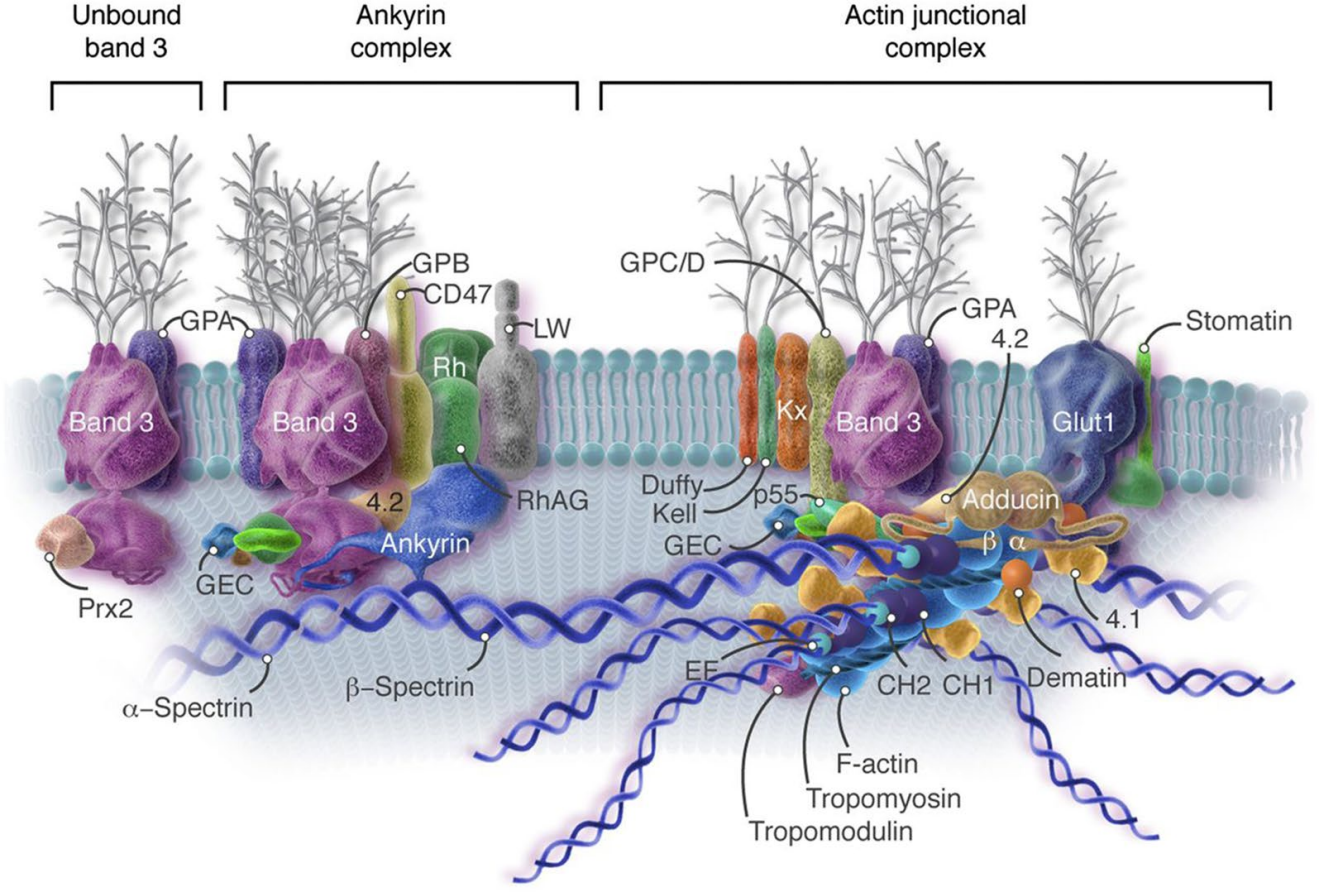
The skeleton structure and membrane protein distribution of RBCm. Reprint with permission from [18]

The RBC membrane (RBCm) consists of phospholipid bilayers, cholesterol, and other components anchored to a two-dimensional elastic network of skeletal proteins by tethering sites on the cytoplasmic domains of transmembrane proteins embedded in the lipid bilayers. The skeleton composed of immobilizing transmembrane proteins presents protection to avoid forming budded shapes [16, 17]. There are more than fifty membrane proteins in the cell membrane [18] (Fig. 1). The four major phospholipids in the cell membrane are asymmetrically distributed. The outer monolayer consists of phosphatidylcholine and sphingomyelin. The inner monolayer consists of most phosphatidylethanolamine and all phosphatidylserine (PS), with minor phosphoinositide constituents[15]. It has been determined that lipid transfer proteins can manipulate the phospholipid composition of the RBCm. The external lipid molecules can change the membrane phospholipids’ arrangement and asymmetry and expose PS at their outer surface. Therefore, macrophages can recognize the engineered RBCs and increase the adhesion of RBCs to vascular endothelial cells [19].

An essential characteristic of RBCs is their excellent deformability allowing them to migrate from narrow capillary systems, such as the splenic sinus. The RBCm is 100-fold softer than a latex membrane of comparable thickness. The membrane has good flexibility, rapid response to applied fluid stresses, and strong ductility, indicating good material potential [16]. Under high mechanical tension, the cell membrane forms pores. The pores remain open for a prolonged time during the high-speed tank-treading-induced stretching and compression process. A higher rate of stretching of the membrane patch can increase the critical areal strain and density of pores [20]. Studies have determined that membrane proteins and cytoskeleton determine the deformability of RBCs [16, 17]; however, this deformability is limited. When the surface area of RBCs increases by 3–4%, it leads to cell lysis [14], suggesting that attention should be given to surface area stability when handling RBCs. Other factors affecting RBCs’ elasticity include hemoglobin concentration and spectral proteins. For example, at a higher hemoglobin concentration, the viscosity of RBCs increases, and elasticity decreases [21].

Additionally, the domain boundaries of the membrane could change with imperfect lateral packing, enhancing membrane permeability [22]. Also, any manipulation that leads to membrane mechanical stability or defective ion transporters could reduce its deformation capacity and accelerate its removal in the cycle. All these factors compromise the ability of the cell to deform and lead to its premature removal from circulation. In addition to other cell membranes, the surface of RBCm carries negative charges. The negative charge could stabilize RBC suspension [21].

### Physiology of RBCs

RBCs have unique physiological characteristics. The life span of RBCs in humans is 120 days, while that in mice is 40 days. The senescent and damaged RBCs are phagocytosed by the mononuclear phagocyte system (MPS) in the spleen and liver. The surface of healthy RBCs expressing CD47 can combine with macrophage SIRPα, providing a strong negative signal for phagocytosis [11, 23]. After the aging or destruction of RBCs, the expression of CD47 decreases actively or passively. Accumulation of cytosolic peroxiredoxin-2 at the inner cell membrane was proposed as a marker of oxidative stress in RBCs. When RBCs are senescent, some changes occur in the membrane markers, like increased externalized PS and decreased CD47 levels [24]. Another unique feature of RBCs is the absence of nuclei and organelles, and mature RBCs expel their nuclei before entering circulation [25]. In mammals, nucleated primitive erythrocytes are found in the circulating blood vessels during the embryonic stage. However, they gradually disappear during their transition into the fetus. They migrate to the liver and produce denucleated erythrocytes [25]. During differentiation, RBCs gradually become smaller through continuous division.

A recent study showed that RBCs can express TLR9, bind pathogens, and accelerate erythrophagocytosis and innate immune activation [26]. Another study also identified four specific classes of precursor erythrocytes by sc-RNA-seq and Gene Ontology enrichment analysis. In addition to developmental differentiation and oxygen-carrying functions, there is a class of precursor erythrocytes with immune relevance. These clusters express serglycin and NF-κB inhibitor alpha, associated with inflammatory cell secretory granules and NF-κB-mediated immune and inflammatory activity [13]. This indicates that red blood cells have potential immune functions.

Another unique physiological feature of human RBCs is the existence of specific proteins, such as Rh and ABO blood group system, on their cell membranes, leading to RBCs in the environment without corresponding antibodies; otherwise, hemolysis occurs [27].

### RBCs in the microenvironment

The external pH environment can change the shape of human RBCs. RBCs form stomatocytes at low pH and schistocytes at high pH [28]. A study showed that high-frequency electric fields can induce the deformation of RBCs. The electrical membrane breakdown could lead to depolarization and hemolysis [29]. When RBCs are removed from physiological conditions and stored at 4 °C, the membrane Na^+^/K^+^ pumps will be inactivated, and phospholipids-rich, CD47-positive microvesicles are produced. RBCs undergo morphological deformations during microcirculation, such as changes in surface area, volume, and sphericity [30].

Various cytokines have different influences on RBCs. Growth factors influence the differentiation of RBCs. The earliest erythroid progenitor cells respond to cytokines, including thrombopoietin, granulocyte-macrophage colony-stimulating factor, IL3, and IL11, especially stem cell factor. Stem cell factor synergizes with erythropoietin (EPO) in proliferating and expanding developing erythroid progenitor cells and may play a crucial role in phosphorylating EPO receptors. Growth factors can affect the differentiation and apoptosis of RBCs. In a mouse model, interferon-gamma can reduce RBC lifespan and inhibit RBC generation by activating macrophages [25]. Many proteins on the RBCm bind specifically to antibodies in the environment. This binding can alter some of the physical and chemical properties of RBCs, such as anti-band 3 binding with major sialoglycoprotein, glycophorin A, reducing the deformability of RBCs [31].

## RBCs and their derivatives in the delivery system

### Advantages of RBCs as delivery systems

The morphological characteristics of RBCs accredit them with a larger surface area and as a type of biofilm. The RBCm comprises phospholipid components and is governed by membrane bending energy. Therefore, due to the negligible bending energy of the skeleton, the shape behavior of RBCs and phospholipid vesicles could be similar. However, Due to RBCs’ physical features, RBCs are considered suitable materials for drug delivery and achieve therapeutic potential.

First, anucleate RBCs provide more room for drugs and can be safely used for genetic modifications [11]. Second, dark red RBCs are easily heated by near-infrared (NIR) light [32]. This property can be used for acoustooptic therapy. Third, the surface of RBCs expresses protein molecules that avoid being engulfed by the immune system, and the survival period is long. Thus, the delivery system can escape immune clearance and release drugs in the body for a long period. Fourth, as RBCs circulate in the blood vessels, they reduce the contact between the drugs encapsulated in RBCs and other substances in the microenvironment, reduce the metabolic clearance factors of drugs, and reduce the off-target side effects of drugs. Because of these unique shape advantages of RBCs, several studies have prepared drug carriers to transport drugs by imitating the shape of RBCs [33, 34]. On the other hand, the RBC biodistribution ends up in the spleen and liver, which can reduce nonspecific and undesirable off-target effects. This character can target immune organs. and RBCs can function as vaccinum. As mentioned above, it is easy to find that different treatments of RBCs lead to different expressions of morphodynamics, which leads to distinct functions. RBCs and their derivatives have different properties, so that they can be used in different directions. The RBC delivery system could change pharmacokinetic and biodistribution characteristics based on these characteristics. They can prolong drug release time, extend the half-life of drugs, reduce immunogenicity, and diminish adverse reactions.

### RBCs as carriers

#### RBCs intracellular drug loading

Many drugs or diagnostic materials are limited by their low bioavailability, short half-life, and circulatory toxicity. On the other hand, RBCs have a long life span, and whether they can behave like a potential delivery system to prolong of drug’s action in the body has attracted researchers’ interest. RBCs, as carriers, can load drugs intracellularly or couple the molecule onto the cell’s surface via protein adhesion. RBC loading demonstrated a sustained release and prolonged the drug’s half-life. To prepare RBC carriers, some methods like hypotonic swelling and hypotonic dialysis [35–39], sonoporation [40], fluidic shear stress [41], electroporation [42], using chlorpromazine (CPZ), and fusion with liposomes [43] have been adopted. The most used method is hypotonic swelling. RBCs have good deformation ability, expanding into spheres in low permeability liquid without rupture. At the same time, the pores open large enough to allow the carrier to enter. RBC can be restored to a double concave disk shape and used as a carrier in the hypertonic environment. A study determined that this method could affect specific characteristics of RBCs but does not affect the lifespan of RBCs or their drug carrier functions [39].

RBCs were first used as DDS for loading enzymes [44]. ERYtech Pharma has produced a product called GRASP (erythrocytes encapsulating l-asparaginase) to treat acute lymphoblastic leukemia (ALL) [45]. Recently, this production completed its phase 2/3 study. The open, randomized, international trial enrolled eighty-five participants. The results showed that GRASP prolonged days of asparaginase activity to 18.9 d to 8.5 d (free l-asparaginase) and reduced the allergic reaction. Even in the allergic population, the anti-allergy effect is better than l-asparaginase monotherapy in the nonallergic population. At the same time, the research team confirmed the effects of the drug on the treatment of advanced pancreatic cancer after chemotherapy.

With the development of bDDS, enzymes and some small-molecule drugs can be loaded into RBCs. In recent studies, trehalose [46], interferon [47], antibiotic [48], hormones [38], pravastatin [49], hydrochloride [37], and ambroxol hydrochloride [35] have been loaded into RBCs. RBC characteristics can achieve novel therapeutic effects compared to traditional chemotherapy.

#### RBCs-hitchhiking

As RBCm has an adhesive effect and the ability to target lung and brain vessels, attaching drug nanoparticles (NPs) to RBCm is another way to deliver drugs in vivo [50]. The drugs adsorb onto RBCm via electrostatic interactions [51], molecular protein anchoring [52, 53], and avidin-biotin coupling [54]. Targeted drugs to the lungs [55–60], spleen [61], tumor sites [54, 62, 63], and bacterial infection sites [64]. This method allowed different materials to disassociate from RBCs to the first organ downstream of the intravenous injection spot. Zhao et al. designed an erythrocyte-leveraged chemotherapy platform that binds doxorubicin (Dox)-loaded biodegradable polymeric NP to RBCm to treat lung metastasis models [65]. The results showed that the drug concentration of the RBC-NP group existed longer in peripheral blood and lung tissue at all times. The survival time was prolonged twice as much as free drugs indicating the RBC-NP has greater efficiency.

The properties of the delivery materials are related to the effects of the delivery system. The size, number, and type of NPs can impact the action of RBCs [66]. Just like the pH of the materials wrapped by the RBCs will affect their release efficiency [67, 68], the zeta potential of RBC-hitchhiked NPs affected the redistribution and circulation effects of the delivery system [55, 69]. A study using a numerical algorithm to predict drug delivery via RBCs-hitchhiking indicated that increased shear rate and NP sizes could facilitate drug release [70].

If the nanoparticles are linked with high affinity to mouse erythrocytes peptide, they can attach RBCs in vivo. A study verified ERY_1_ peptide can increase binding to the erythrocyte membrane. This method of attachment avoids operational damage to RBCs in vitro, completely retaining the biomarkers and biocompatibility of RBCs. They found that this delivery system can reduce the levels of TNF-α and interleukin-6 (IL-6) in vivo, indicating RBC-based delivery system might have some immunotherapy potential [63].

#### Engineered RBCs as vaccines

**Fig. 2:**
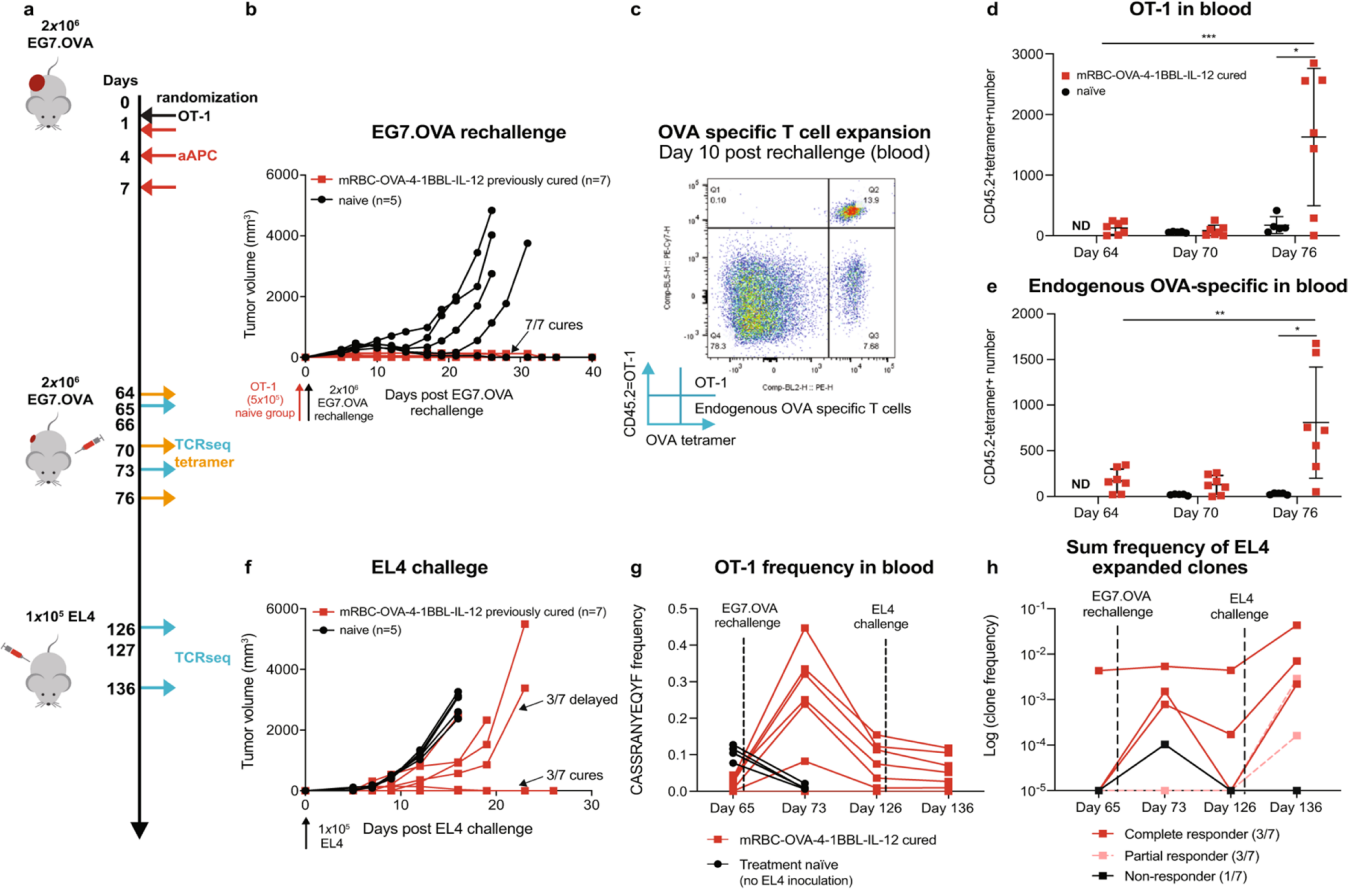
RBCs as APCs promote immune memory and epitope spreading, and harness endogenous T cells. **a** mice with EG7.OVA tumors were first treated with naïve OT-1 cells, and dosed with mRBC-OVA-4-1BBL-IL-12. Then the survivors were rechallenged on day 66 with EG7.OVA. The control group chose age-matched naïve mice treated on day 65 with OT-1 cells 1 day before challenge with EG7.OVA cells. **b** All previously cured mice rejected EG7.OVA rechallenge. **c** OT-1 and endogenous OVA-specific T cells both expressed in peripheral blood 10 days after EG7.OVA rechallenge. **d**, **e** OT-1 and endogenous OVA-specific T-cell numbers in peripheral blood were significantly high expression 10 days after rechallenge. **f–h** mRBC-OVA-4-1BBL-IL12 promotes epitope spreading. Reprint with permission from [61]

RBCs have a spleen-homing effect, so the delivery system can be prepared as a vaccine to target antigen-presenting cells (APC) and enhance antitumor immunity [71, 72]. Erythroid precursor cells undergo gradual enucleation during differentiation. This character allowed genetic modification in erythroid precursor cells without incorporating the modified genes into the vaccine. Some researchers attached nanoparticles binding specific antigens to the surface of RBCs to target the spleen by adjusting the particles on the surface. This delivery system improved central memory T cells and reduced Treg cells, enhancing specific cellular and humoral immunity. It plays a preventive immune role in tumors [61]. Furthermore, researchers modified RBCs to APCs to activate tumor-specific T cells by expressing major histocompatibility complex I (MHC I), the costimulatory ligand 4-1BBL, and IL-12 on the surface of RBCs [11]. The results showed that these engineered RBCs reduced the circulating toxicity of 4-1BBL and IL-12 and generated memory immunity and epitope diffusion (Fig. 2). Using RBCs as a vaccine was a successful attempt and can specifically target different tumor immunotherapies by altering the tumor antigens linked to the MHC I.

### RBC-derived vesicles in delivery system

RBCm or erythrocyte ghosts are pale RBC membranes with no or minimal hemoglobin. The size of RBCm is similar to that of original RBCs [73]. RBC ghosts had higher PS and were easily swallowed by phagocytes in vitro [74]. They were easy to prepare and could load more poorly stable, fragile, or potentially immunogenic agents. Another significant advantage of RBCm is that their size can be compressed. The micrometer-level size of RBCs could limit them through vessel walls or reticuloendothelial (RES). A study indicated that particle size affected lifespan and accumulation in the liver of RBCs in circulation. Generally, the larger the cell diameter, the shorter the cell life span, more significant the accumulation in the liver [75]. In a recent study, researchers reported that the membrane stiffness of micro-RBC-derived vesicles (RDVs) is higher than that of RBCs by approximately 28−62%. In this case, there was a reduction in the deformation capability of micro RDVs for effective splenic passage and aggregation. However, nano RDVs do not have this issue [76]. Therefore, researchers reduced the RBC volume and retained their biocompatibility and other characteristics. RDVs can solve this problem. The standard strategies to prepare nanoscale erythrocytes include sonication and extrusion. The diameter of RDVs can reach < 200 nm. It has a half-life 2.5 times longer than nanoliposomes [73, 76–78]. From confocal microscopy, we can demonstrated that the loaded drugs can extravasate via the tumor vessel and penetrate deeply into the tumor[74]. Simple methods, like shear force, can get nanoscale vesicles [41, 79, 80]. The nano RDVs have intact membrane proteins and glycolipids, exhibiting better stability than single liposomes. They have an endogenous nature and low immunogenicity. Therefore, many researchers have used RBCm in single drug delivery and new therapeutic strategies, such as thermotherapy, photodynamic immunotherapy, and sonodynamic therapy[76, 81–84]. RBCm has been used as nanocarriers since the mid-1990s [85]. The strategies for applying RBCm to drug delivery will be more diversified. RBCm can deliver drugs alone or combined with NPs or other biomembranes.

#### Simple RBCm loading

RBCm plays a carrier role via different kinds of disposal. The most loaded disposal is the anti-cancer drugs [86]. Hsieh et al. designed a drug-loaded RBC membrane shell. They added an organic phase (perfluoro-n-pentane, C5F12) to achieve acoustic vaporization to this vesicle. By putting the organic phase into a continuous aqueous phase containing RDV and being broken up by sonication, the RBCm can cover and stabilize it. At the same time, antitumor drugs can be loaded into it. The average size of this vesicle was 1.7 μm. Most membrane proteins were found on this vesicle, suggesting that the sonication procedure did not cause much loss of proteins from the RBC membrane, and biocompatibility was preserved [87]. However, there is a study using the long circulation character of RBCm to wrap charge-reversible polyplexes of siRNA [88, 89]. Wang et al. first determined the proper proportion of bovine serum protein (BSA) and siRNA to structure charge-reversible polyplexes (RPs). The RBCm was extruded at 200 nm, and the RPs were cloaked. When the pH dropped to 5, the membrane was ruptured because of the proton-buffering effects and released siRNA for sequence-specific target gene knockdown. These results showed that RBC-RP could avoid decreased macrophage phagocytosis efficiency.

**Fig. 3:**
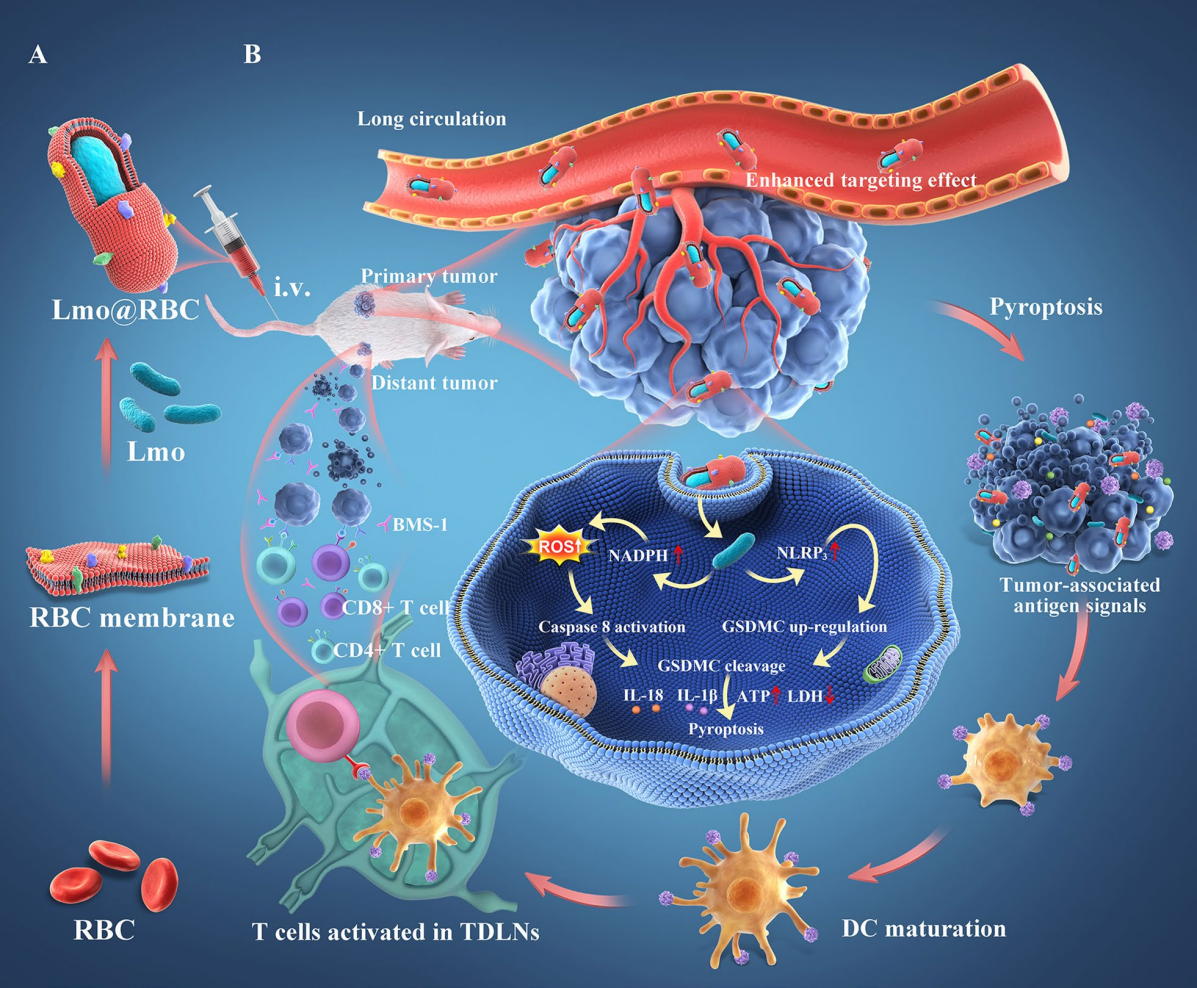
RBCm camouflages Lmo to kill cancer cells. Reprint with permission from [90]

Natural polymer compounds, such as siRNA and living bacteria, can also be loaded into RBCm. *Listeria monocytogenes* (Lmo) have been loaded by extrusion with RBCm [90]. The size of Lmo@RBC increased by 200 nm compared to that of simple Lmo. Both CD47 and anti-Lmo monocytogenes were expressed on the Lmo@RBCs. Using RBCm, Lmo could circulate in the blood for a long time until it reached the tumor site. This caused a considerable accumulation of tumors because of the hypoxic microenvironment of the tumor sites suiting this anaerobic Lmo colonization (Fig. 3). This differs from the simple wrapping of complete RBCm on the surface of NPs, which is used in most studies. Wu et al. separated the endogenous proteins and lipids from natural RBCm to “disassembly and reassembly” to produce a new RBCm-NP [91]. It is a green technology that eliminates hazardous substances, prevents health impacts in the design process, and preserves structural integrity [92]. In this study, the separated membrane proteins were added to the film by hydration method to synthesize IR780@rRBC NPs. Compared to the RBCs directly loaded with the drug, the IR780@rRBC NPs were more uniform and spherical, and the particle size was smaller (IR780@RBC: 156.4 ± 16.8 nm vs. IR780@rRBC NPs: 80.28 ± 12.4 nm). From decreasing toxicity, increasing stability, prolonging circulation, and enhancing photothermal therapy (PTT), the IR780@rRBC NPs performed better than IR780@RBCs. The researchers deduced that this was because of the uniform distribution of IR780 in the vesicles.

#### RBCm camouflage nanoparticles

Many researchers used a cell membrane coating to maintain the relative stability of nanoparticles during circulation in a complex blood environment. RBCm as camouflage is used to extend circulation and immune escape, while the NP core contributes to high drug loading. The coated nanoparticles include lipid multichambered nanoparticles [93, 94], metal nanoparticles[10, 68, 95–98], polymers such as poly(lactic-co-glycolic acid) (PLGA) [99, 100] or polyethylene glycol (PEG) [101, 102] and some new nanomaterials like boron nitride nanospheres (BNNSs) [103], albumin[104] and so on [105–107]. From these results, we find that RBCm can improve the stability of NPs and avoid anaphylaxis via injection. All studies showed that RBCm-NPs have a better effect on tumor treatment in vitro and in vivo. The immune evasion ability increased by 50–60% [106]. In addition, the blood circulation of drugs was doubled [97].

From the perspective of RBC morphology. A study used Ca(OH)_2_ microparticles with a biconcave discoidal morphology as templates and coated RBCm to build an RBC-mimetic micromotor (RBCM) [12]. The biconcave discoidal morphology provided good deformability, allowing the micromotor to easily pass through capillaries. RBCm can help micromotor escape immunity and phagocytosis. The pharmacokinetics showed that the plasma concentration of RBCM is higher than a free micrometer, and the imaging contrast of the RBCm was enhanced at the tumor site, implying the existence of more RBCm in the tumors. RBCm-coated elastic poly(ethylene glycol) diacrylate hydrogel nanoparticles simulating dynamics have been developed with good deformation ability [108, 109].

**Fig. 4:**
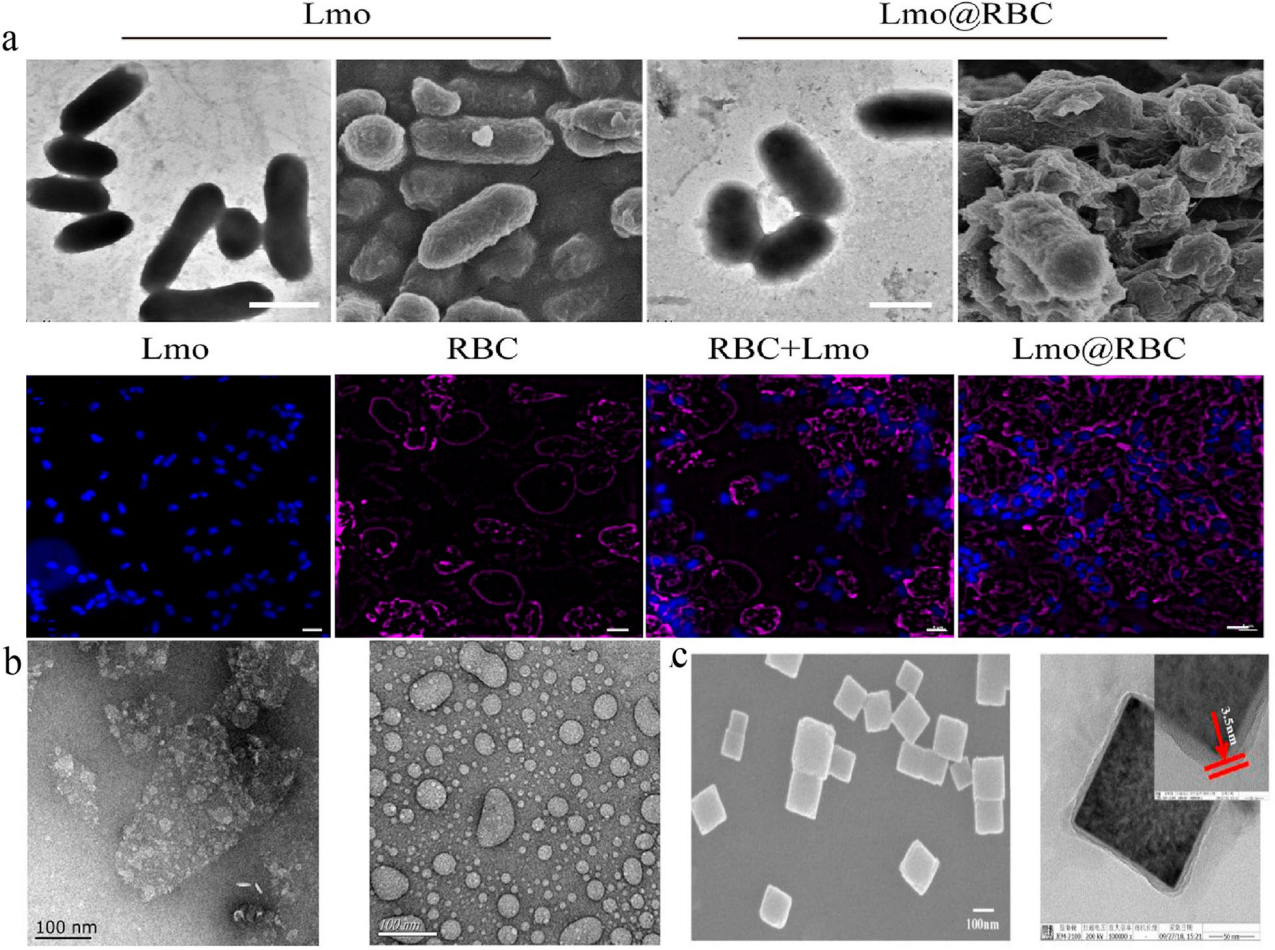
Morphology of nonspherical RBCm camouflage. **a** Transmission electron microscope, Scanning Electron Microscope and Confocal laser scanning microscope of Lmo and Lmo@RBC. **b** Transmission electron microscope of RBCm modified on the surface of GO. **c** Transmission electron microscope of RBCm@prussian blue. Reprint with permission from [90, 106, 111]

In most studies, the RBCm is used to coat spherical NPs. But it can modify non-spherical or two-dimensional materials [10, 110] (Fig. 4). For example, a study used RBCm to camouflage on the surface of two-dimensional graphene oxide (GO) nanosheets for tumor chemotherapy [111]. The RBCm is adsorbed on the surface of GO by incubation, and Dox is attached on RBC-GO through p-p conjugation and electrostatic adsorption. The results verified that Dox-RBC-GO could improve the stability and biocompatibility of GO nanosheets and demonstrate better antitumor efficacy and lower toxicity. Additionally, RBCm combined with NP possesses the features of real RBCs with a similar size and biconcave discoidal morphology.

From the perspective of RBC physiological action. Some studies revealed that RBCm camouflaged NPs showed superior PTT efficacy compared with NPs and PEGylated NPs [112, 113]. RBCm can solve the issue of poor biocompatibility and biodegradability of some potential nanophotothermal conversion materials. Many studies explored the effects of the delivery system using RBCm to camouflage nanomaterials loaded with photosensitizers or photothermal agents in photothermal or photodynamic therapy [95, 114–126]. Zhang et al. mixed RDV and human hair nanoparticles (HNPs) and subjected them to ultrasonic treatment to encapsulate the HNPs to exert the photothermal effect of melanin. To enhance the targeting ability, they functionalized DSPE-PEG-cRGD to the surface of RBCms. The size of HNP@RBCm was 93.51 nm, and the membrane protein, CD47, was well preserved. These results indicated that RBCms can load HNPs and preserve their biocompatibility [127]. However, the extra surface coating might inhibit heat dissipation, researchers encapsulated the photocatalyst titanium and photothermal agent in RBCm to design light signal-activated bionic nanocapsules [128]. The vesicle can be cracked under specific photocatalysis for photosensitization.

#### Hybrid membrane as a delivery system

RBCm can fuse with other biofilms and play a complex role. RBCm has an important position in membrane fusion functionalization strategies. Other membranes, like tumor cell membrane [129, 130], platelets [131–133], or liposomes [134, 135], have been fused with RBCm. Based on the same principle, combining fusion membranes with NPs might endow more functions. The disease-related cell membrane is fused with RBCm to enhance targeting effects. Head and neck squamous cell carcinoma [130], human breast cancer cells [136, 137], and liver cancer cells [138] have been demonstrated that can fuse with RBCm. Xiong et al. created a hybrid biomimetic fused ovarian cancer cell membrane with RBCm to mimic Fe_3_O_4_ magnetic nanoparticles coated with indocyanine green (ICG). This delivery system can specifically target tumor sites and perform synergistic PTT. RBCm ensured that the magnetic field existed longer in the circulation and enhanced immune escape ability [139].

To achieve more drug aggregation at the tumor site, researchers used the characteristics of the platelet membrane that can recruit the combined reaction at the vascular endothelial injury site. The fusion of platelets and RBCm and the encapsulation of photothermal polymers can target tumor microvessels, promote immune escape, and prolong circulation time. Compared with a single NP, the particle diameter of the delivery system wrapped by biofilm increased by 40 nm. Pharmacological results and distribution in vivo suggested that RBCm has better immune escape ability than the platelet membrane, characterized by longer internal circulation and less distribution in the liver and spleen than single platelet biomimetic nanoparticles. After near-infrared light irradiation, the NP of the platelet-red cell fusion membrane showed the best distribution concentration [132]. Incorporating thermosensitive lipid (TSL) membrane into RBCm and MCF-7 cancer cell membrane can enhance chemo-/photothermal combined tumor therapy [138]. Huo et al. used this hybrid membrane vesicle to coat Dox-loaded hollow gold nanoparticles. The results showed that this vesicle exhibited better antileakage and higher NIR responsivity. The accumulation of Dox at tumor sites increased by four times due to RBSm. In addition to using tumor cell membranes for target homing, other disease-related cell membranes can be part of the fusion cell membrane. For example, Yu et al. fused fibroblast-like synoviocytes and RBCm to camouflage Prussian blue nanoparticles loaded with an anti-rheumatoid arthritis compound to treat rheumatoid arthritis [140].

#### RBC budding vesicles as a delivery system

**Fig. 5:**
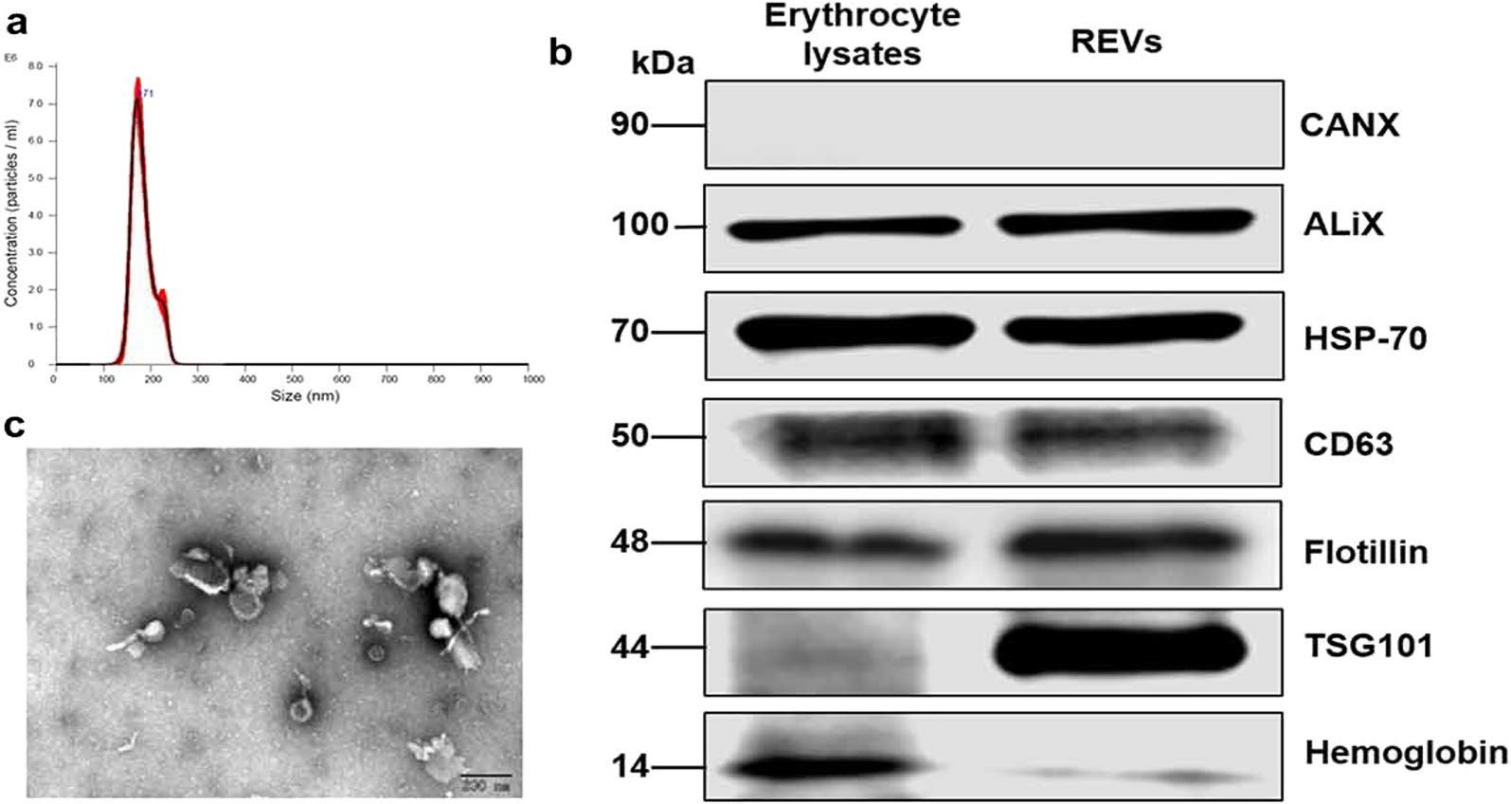
The difference of surface markers between EVs and normal RBCs. **a** The size distribution of EVs. **b** Different protein expression between RBCs and EVs. **c** Morphology of EVs under transmission electron microscope. Reprint with permission from [141]

RBC-derived biofilm-like budding vesicles have been used as a delivery vehicle. Erythrocyte vesicles (EVs) have low immunogenicity and cytotoxicity. The biomarker of EV is similar to normal RBCs, but TSG101 is more enriched [141] (Fig. 5). Because of membrane proteins, EVs are softer than pure lipid liposomes, demonstrating their potential for flexibility. However, the mechanism of vesicle formation is unknown. Sorkin et al. demonstrated that different temperatures or incubation times might influence vesiculation mechanisms [142]. Under low temperature conditions (22 °C), the EVs exhibited a higher bending modulus than that under physiological temperature (37 °C) and extremely low temperature (4 °C). The proposed mechanism might include protein aggregation and cytoskeleton-induced buckling. Another study used CaCl_2_/EDTA to induce the budding of RBCs. The harvested EVs were linked to Dox, verifying their antitumor effects [143]. The final diameter of the combination is 487 nm, which is larger than that of the EVs obtained by extrusion but is still much smaller than RBCs. The membrane of EVs has some differences from that of RBCs. The EV membrane expresses less Hb than RBCs. Additionally, EVs can cause an acute innate immune response. However, functionalization of nano RDVs with folate or herceptin can reduce the cytokine response [144]. Also, decorating the membrane surface of EVs can improve the pharmacokinetics and concentrate drugs in the targeting site at > 50% higher than those without modification [145]. In vivo results showed that EV-based carriers use lower doses of drugs to achieve antitumor effects comparable to or even superior to those of high-dose free drugs. Interestingly, this excellent antitumor efficiency may be due to the different delivery pathways of EVs. A study found that Dox linked with RDVs is released into the lysosome instead of the nucleus, activating the reactive oxygen species (ROS) system. However, the biodistribution of engineered EVs was not well explained in this work, and we cannot deduce whether they have better biocompatibility [143]. Furthermore, there are other methods to generate EVs. For example, there are researchers who used HlyA-treated erythrocytes, which increased intracellular calcium concentration and activated purinergic receptors to secrete EVs. Additionally, EV delivery systems have been used in some diseases related to erythrocytes, such as malaria. Xu et al. produced EVs by RBCs and *Plasmodium*-infected RBCs (pRBCs). The size of these EVs was 175–200 nm. pRBCs produced more EVs than normal RBCs. From flow cytometry results, these two kinds of EVs showed a better ability to combine with pRBCs than normal RBCs. Although the mechanism is unclear, the results showed that RBCs and pRBCs could internalize the pRBC-EVs at a higher efficiency. Based on this, researchers loaded antimalarial drugs—atovaquone and tafenoquine—into pRBC-EVs. In addition, it performed more efficiently in inhibiting the growth of *P. falciparum *in vitro [146].

## Application of RBC delivery systems

### Tumor treatment

RBCs have excellent biocompatibility and long circulation characteristics; they have been used to deliver antitumor drugs. Many antitumor drugs have defects, such as short half-lives, low bioavailability, and easy clearance by the monocyte-phagocyte system [37, 62, 147]. There is no choice but to increase the dosage of drugs to achieve the therapeutic effects, thus increasing the risk of dose-dependent side effects. Additionally, the treatment of tumors requires a relatively long period. The characteristics of RBCs can enhance the antitumor effects.

Recently, some new treatment strategies, such as sonodynamic therapy, photodynamic therapy (PDT), PTT, and magnetic targeting, have received increased attention. However, these strategies need some mediums to achieve the effects, such as photosensitizers, photothermal, and magnetic particles. These media have low biocompatibility, which limits their application in vivo [148, 149]. RBCs can camouflage these media as biologically active cells to make these strategies more practical. RBCs and their derived vesicles have been combined with common mediums, such as gold nanorods (AuNRs) [95, 112], Prussian blue/manganese dioxide nanoparticle (PBMns) [150], ICG [123, 151, 152], iron oxide [12, 153–155], and magnetic mesoporous silica nanoparticles (MMSNs) [83]. With the RBC delivery system, some novel tumor treatment methods could be popularized.

#### Optimize acousto-optic therapy

**Fig. 6:**
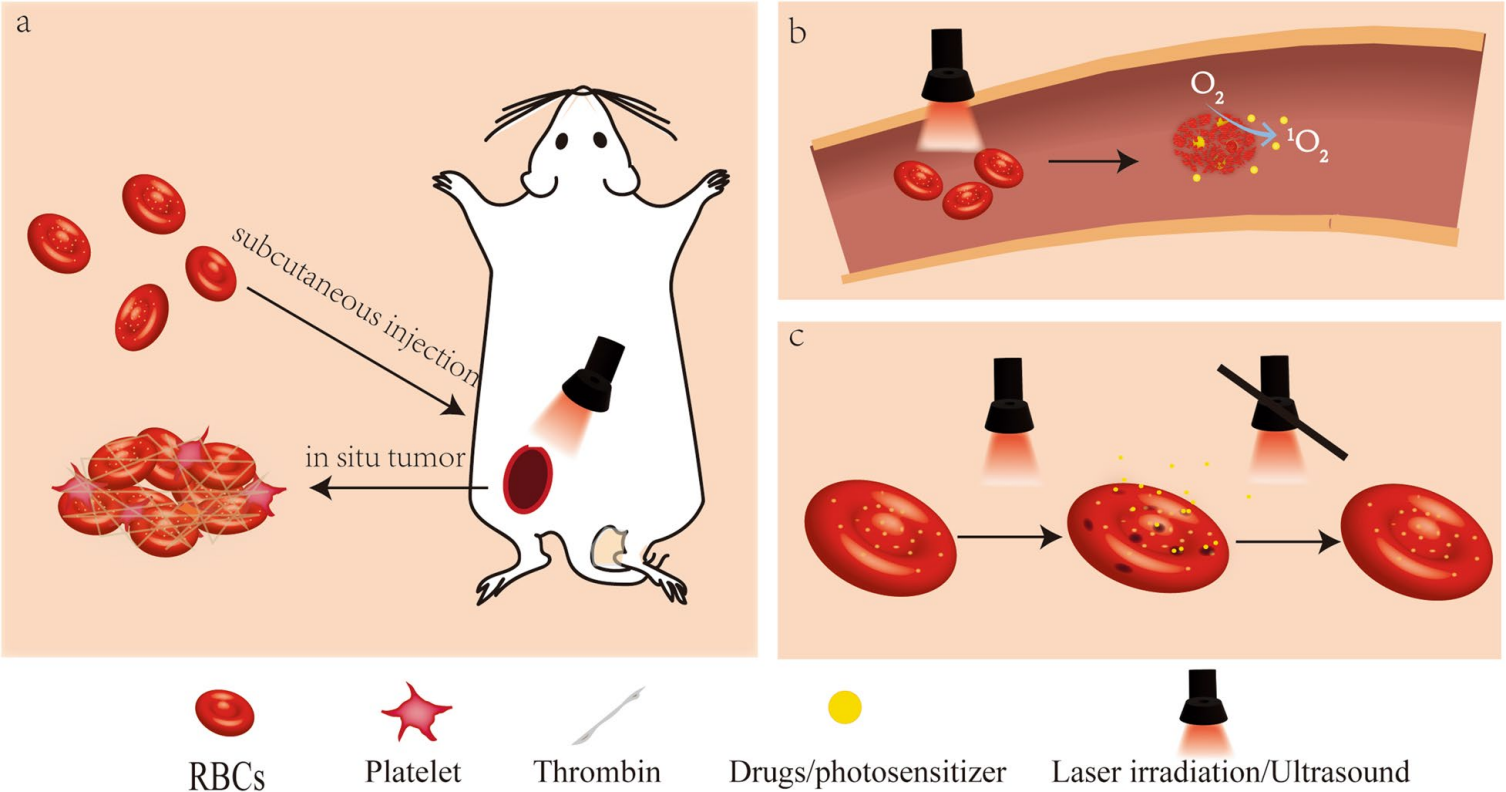
Several application methods of RBCs in optimizing acoustoptic therapy. **a** RBCs-gel formed at the tumor site through coagulation pathway and release drugs through NIR irradiation [32]. **b** RBCs can be destroyed by laser in blood vessels and release drugs by encapsulating photosensitizers [134, 157]. **c** RBCs can achieve laser-controlled drug release through specific photosensitizers (ICGs) [151]

Due to the deep-red color of RBCs, near-infrared light can react with RBCs [32]. Some researchers have used optical absorption and photosensitizers to burst RBCs and achieve precise treatment. (Fig. 6) One research loaded vitamin B_12_, taxane, and Cy5 antennae into RBCs to exert tumor phototherapy [156]. The Cy5 antenna can sensitize the conjugate to far-red light, circumventing hemoglobin’s intense light-absorbing properties at 350–600 nm. As VB_12_ is membrane impermeable, photolysis separates the taxane from the B_12_ cytoplasmic anchor, achieving targeted antitumor effects. The RBC carrier can prolong the circulation time of drugs and phototherapeutic efficacy. The fluorescence of RBC@Cy5-B_12_-TAX maintained 53 ± 5% of its fluorescence after 90 min while free B_12_ ≡ Cy5 extravasated from blood vessels in 5 min. However, the study suggested that engineered mice RBCs (mRBCs) were more fragile and susceptible to lysis than human RBCs in vitro, so mRBCs are unsuitable for RBC delivery efficiency verification. Another use of bursting RBCs to achieve the therapeutic aim strategy is installing photoactivatable molecular triggers on the RBCm to burst the RBC vehicle under laser irradiation. The application of this strategy can burst RBCs at specific sites. This delivery system is effective at exhibiting long-term stability in systemic circulation and releasing its cargo in a controlled and precise manner. A study used this cell-based vehicle that was covalently conjugated with 2-(1-hexyloxyethyl)-2-divinyl pyropheophorbide-α (HPPH) as photoactivatable molecular triggers [157]. Thrombin (Th) and tirapazamine (TPZ) are loaded into RBCs to achieve thrombosis-induced starvation therapy. This vehicle (Th/TPZ@HRBCs) showed that the leakage of thrombin is slow within 25 h but undergoes an increase of over 90% after laser irradiation treatment. In an in vivo trial, the Th/TPZ@HRBCs with laser irradiation showed a sharp increase at 6 h post-injection. The blood regions were blocked for at least seven days without substantial recovery. Moreover, because of the intrinsic blood circulation property of RBCs, the encapsulated thrombin was stuck in vessels.

Other researchers used RBCs to act as photosensitizers enabling photoablation. Researchers have used RBCs as photosensitizersto enable photoablation (PA) of tumors [32]. The theory of this study relies on subcutaneously injected RBCs triggering physiological signals, such as platelets and thrombin, to form hydrogels in situ. The immune adjuvant imiquimod was attached to the RBC membrane. When RBC-gel was heated to burn tumors and release tumor-associated antigens, imiquimod was released into the tumor-draining lymph node. There are other ways to use lasers to release drugs loaded in RBCs. Shao et al. fabricated a remote laser-controlled drug delivery system [151]. By loading the photosensitizer ICG and insulin into RBC, ROS can be generated under laser irradiation and open the RBC phospholipid bilayer. Conversely, the system will close when the ROS is scavenged without laser irradiation. These studies showed that with ingenious use of characteristics of RBCs, active or passive lysis of RBCs achieves the efficacy of targeted drug release.

Ultrasound can be used in RBC delivery systems for tumor treatment. A liposome and RBCm fusion carrier loaded a universal sonosensitizer and an antitumor drug. This delivery system can generate ROS to oxidize the unsaturated phospholipids in the hybrid nanovesicle under ultrasound stimulation. This delivery system can achieve a better-controlled release of drugs [134]. Similarly, the C5F12-RBC delivery system can achieve acoustic vaporization. Under high-intensity focused ultrasound, the C5F12 would be vaporized, destroying the RBCm and releasing the drug[87].

The RBC delivery system can help the cooperative treatment of multiple therapies [121]. RBCm were used to load gold nanorods and glucose oxidase to combine PTT and glucose-consuming starvation therapy for colorectal cancer therapy [95]. This NP can aggregate at the tumor site and be triggered under NIR irradiation. With the membrane rupture, the drugs will be released and deplete endogenous glucose to restrict the energy supply to tumor cells. Meanwhile, the heat shock proteins will express and inhibit the deficiency of ATP to enhance the efficacy of PTT.

#### Optimize magnetic targets therapy

RBCs loaded with drugs exhibit longer circulation time. It is convenient to navigate RBCs to targeted sites and release drugs. A noninvasive and harmless magnetic field can target the drug delivery system to the target area. A study demonstrated iron oxide magnetic nanoparticles (IONPs) with an imaging agent (CdTe QD) and antitumor drugs (Dox) into erythrocytes. This system can achieve precise transport of the cargo under ultrasound. The uneven distribution of the encapsulated magnetic nanoparticles within the RBC micromotor under the applied magnetic field drives the movement. Such asymmetric particle distribution inside the RBC motor resulted in an acoustic pressure gradient in the fluid, causing movement [153, 158]. Wang et al. used an applied external magnetic field. In this system, the RBC is attached with IONPs coated with chlorine e6 (Ce6) and loaded with Dox to tumor sites [159]. The system coated with RBC (Dox@RBC-IONP-Ce6-PEG) showed enriched accumulation in 12 h via fluorescence imaging and tumor homing. Meanwhile, the free Dox@IONP-Ce6-PEG showed body weight loss, while no such effect was observed in the group treated with Dox@RBC-IONP-Ce6-PEG. This indicated that RBC-based treatment combined with magnetic effect could achieve targeting treating effects and reduce the side effects of chemotherapeutic agents.

### Bioimaging

Like antitumor drugs, many imaging agents have defects like easy deactivation and poor targeting. Based on this, the RBC delivery system can be a contrast agent with potential in the imaging field [76, 154]. Unlike other polymer materials that induce immune responses, the RBCm demonstrates biocompatibility and adsorbs little proteins when exposed to human plasma. RBCm can protect targeting ligands on NPs’ surfaces, like upconversion nanoparticles (UCNPs), from attaching long-lived “protein corona” [160]. UCNPs camouflaged with RBCm and modified with targeted molecules can realize PET imaging with short half-life radionuclides to visualize breath tumor imaging [161]. Another research used RBC-loading ICG and crosslinking UCNPs to design an RBC-based probe (RBCq). This probe can retain at the tumor site for 4 h and showed a superior signal-to-noise ratio at the optimal time window. It can guide precise tumor resection under an 808 nm laser irradiation [84]. RBCm can load IR780, which is hydrophobic, has high crystallization, and plays a role as a fluorescence imaging/photoacoustic imaging dual model imaging probe. Superparamagnetic magnetic nanoclusters (MNCs) loaded with RBCm can be used in T2-weighted magnetic resonance imaging (MRI) [124]. This research demonstrated that RBCm could improve the targeting efficiency of tumor imaging.

### Immunotherapy

Some RBC delivery systems have shown immunotherapy effects in vivo. This effect is reflected in different aspects according to different treatment methods. Some researchers used galactose-modified RBC to target tumor-associated cells (TAMs) to reverse the TAM phenotype from M2 to M1 [162]. Using this carrier can improve the tumor immune microenvironment and promote tumor immunotherapy. Some researchers used RBC to load immune stimulants, core-shell metal ion-drug nanoparticles, or living bacteria that may have severe systemic inflammation [6, 32, 90, 163]. This indicated that RBCs are safer, more efficient, and have more accurate effects during immunotherapy. Another way to add tumor antigens onto the RBCm is to fuse the cancer cells with RBCm by sonication and membrane extrusion (nano-Ag@RBC) [164]. The damaged RBC can be rapidly cleared and present tumor antigen. The in vivo results showed that macrophages, DCs, NK cells, B cells, CD4^+^, and CD8^+^ T cells were effectively activated. However, the induced immune response had little effect on inhibiting the tumor growth because of PD-L1, so this vaccine must be used in combination with anti-PD-1 preparation. The researchers proposed that combining RBCs and resected tumor tissue cells can develop a personalized and precise tumor vaccine.

### Other aspects

The malaria parasite attacks the RBCs; therefore, researchers used EVs to deliver antimalarial drugs by fusing with the infected RBCs. The malaria parasite can be exposed to high drug concentrations to achieve an effective insecticidal effect [146]. EVs derived from RBCs are rich in phosphatidylcholine, possibly conducive to fusion with infected RBCs.

Loading special materials can also help store RBCs. Trehalose, an excellent active protective agent, can be loaded into RBCs and is essential in protecting RBCs against freeze-drying damage [46]. This is based on the impermeability of RBCm to trehalose.

RBC delivery system is often used for anti-infection and anti-inflammatory treatment. It is most commonly used as a loading hormone for anti-inflammatory treatment [59, 165, 166]. Some researchers have found that RBC-hitchhiked ivermectin (IVM) exhibited low plasma concentration after oral administration and enhanced the delivery of IVM to the lungs, improving the accumulation of IVM in the lung tissues, inhibiting the inflammatory reaction, and reducing the progression of acute lung injury as observed in coronavirus disease 2019 (COVID-19) [55]. Notably, during *Escherichia coli* (*E. coli*) infections, a single type of cell membrane cannot meet the detoxing requirements facing multiple toxins. Therefore, a study developed a polymyxin B (PMB)-modified, RBC-mimetic hybrid liposome (P-RL) to anchor to *E. coli* and neutralize endotoxins and exotoxins from the toxin fountainhead [135]. In this way the detoxification efficiency has been improved, and the detoxification spectrum of existing antiviral systems has been expanded.

Adeno-associated virus (AAV)-mediated gene therapy is a promising therapeutic method, but it is subjected to multiple, high-dose administration and high immune response. RBC delivery system can solve this problem by anchoring AAV to RBCm and predominantly delivering to the lungs. RBC-anchored AAVs showed a four to five-fold enhancement in target gene expression in the lungs compared to free AAVs. AAV particles are sheared-off and deposited in the lungs when RBCs squeeze via the narrow lung capillaries. Meanwhile, this hitchhiking can reduce AAV neutralization by antibodies [56].

RBCm coating can sort cells. A study suggested that RBCm can effectively weaken the adsorption of nonspecific proteins, thus retaining the antibodies modified on the magnetic beads and improving the capture efficiency of target cells. By grafting different antibodies on the erythrocyte membrane, its carrier can select specific cells in peripheral blood. One study is to isolate fetal nucleated RBCs (fNRBCs) by connecting CD147 on the RBCs membrane to noninvasive diagnosis of early pregnancy. More than 90% of target cells were separated from the nanoparticles, and the enhancement purity was about 90% [167]. There are other fields that RBC delivery system can play a crucial role, such as blood sugar and lipids [49] and the treatment of cardiovascular diseases [168, 36]. A fully automated process achieved more efficient and rapid preparation of RBC delivery vesicles [165]. There is no doubt that the RBC delivery system has a broader application that is yet to be explored.

## Current defects and prospects

RBCs have many advantages in the delivery system. The most commonly used ones are their ability to prolong release time and evade immune phagocytosis. But perhaps utilizing the homing effect or immune effect of RBCs is a potential application value in the future.

However, in the process of producing RBC-based drug delivery systems, RBC deformability can be changed. First, various extrusion and other operations during the production process will change the film’s mechanical properties. The change in the volume and concentration of the cell contents affect the viscoelasticity of the cytoplasm and affect cell dynamics. Any modification in the cell membrane surface will affect the flow behavior of cells in vivo [169]. The expression of PS in RBCm induces clearance. Susceptibility to stress-induced PS exposure during in vitro preparation and CD47 loss causes a considerable fraction of RBCs to be susceptible to being removed after transfusion, leading to low deficiency efficiency [170].

Many attempts have focused on RBC delivery systems in various biomedical fields. However, there are few successful applications of this system in clinical practice. The limitations focus on low productivity and strict transportation and storage conditions. Hopefully, there are many studies that attempt to solve these problems. Generating RBCs from human-induced pluripotent stem cells (hiPSCs) is currently the most promising way to produce RBCs in vitro [171]. A new study revealed that hiPSCs generated from hematopoietic stem cells especially peripheral blood sources would be a good option for generating RBCs in vitro [172]. The technology of RBCs preservation is also constantly advancing. Trehalose [173], pre-freeze oxidation [174], and liposome [175] have been determined to have a good effect on freeze drying of RBCs. However, the problem of potential hemolytic and thrombus risks also needs to be addressed before clinical application. There is still a long way to go before the RBC delivery system is prepared on a large scale and enters the clinic.

## Data Availability

Not applicable.

## Abbreviations

RBCs: Red blood cells
bDDSs: Biological drug delivery systems
RBCm: The membrane of RBCs
PS: Phosphatidylserine
MPS: Mononuclear phagocyte system
IL-3/11/12: Interleukin-3/11/12
EPO: Erythropoietin
NIR: Near-infrared
GRASP: Erythrocytes encapsulating l-asparaginase
ALL: Acute lymphoblastic leukemia
NPs: Nanoparticles
GO: Graphene oxide
ELeCt: Erythrocyte-leveraged chemotherapy
APC: Antigen-presenting cells
MHC I: Major histocompatibility complex I
IL-6: Interleukin6
RES: Reticuloendothelial
RDVs: RBC-derived vesicles
BSA: Bovine serum protein
RPs: Reversible polyplexes
Lmo: Listeria monocytogenes
PLGA: Poly(lactic-co-glycolic acid)
PEG: Polyethylene glycol
BNNSs: Boron nitride nanospheres
PTT: Photothermal therapy
HNP: Hair nanoparticles
RBCM: RBC-mimetic micromotor
ICG: Indocyanine green
TSL: Thermosensitive lipid
Dox: Doxorubicin
FLSs: Fibroblast-like synoviocytes
EVs: Erythrocyte vesicles
pRBCs: Plasmodium-infected RBCs
SDT: Sonodynamic therapy
PDT: Photodynamic therapy
PTT: Photothermal therapy
AuNRs: Gold nanorods
PBMns: Prussian blue/manganese dioxide nanoparticles
MMSNs: Magnetic mesoporous silica nanoparticles (MMSNs)
mRBCs: Mice RBCs
HPPH: 2-(1-hexyloxyethyl)-2-divinyl pyropheophorbide-α
Th: Thrombin
TPZ: Tirapazamine
PA: Photoablation
TAAs: Tumor-associated antigens
ROS: Reactive oxygen species
IONPs: Iron oxide magnetic nanoparticles
MF: Magnetic field
Ce6: Chlorine e6
UCNPs: Upconversion nanoparticles
RBCq: RBC-based probe
MNCs: Magnetic nanoclusters
MRI: Magnetic resonance imaging
TAMs: Tumor-associated cells
COVID-19: Coronavirus disease 2019
IVM: RBC-hitchhiked ivermectin
PMB: Polymyxin B
AAV: Adeno-associated virus
fNRBCs: Fetal nucleated red blood cells
hiPSCs: Human-induced pluripotent stem cells

## References

[1] Wu H-H, Zhou Y, Tabata Y, Gao J-Q (2019). Mesenchymal stem cell-based drug delivery strategy: from cells to biomimetic. J Control Release.

[2] Ghosh B, Biswas S (2021). Polymeric micelles in cancer therapy: state of the art. J Control Release.

[3] Muggia F, Hamilton A (2001). Phase III data on Caelyx in ovarian cancer. Eur J Cancer.

[4] Gianni L, Mansutti M, Anton A, Calvo L, Bisagni G, Bermejo B (2018). Comparing neoadjuvant nab-paclitaxel vs paclitaxel both followed by anthracycline regimens in women with ERBB2/HER2-Negative breast cancer-the evaluating treatment with neoadjuvant abraxane (ETNA) trial: a randomized phase 3 clinical trial. JAMA Oncol.

[5] Zhu Y-S, Tang K, Lv J (2021). Peptide-drug conjugate-based novel molecular drug delivery system in cancer. Trends Pharmacol Sci.

[6] Dai J, Wu M, Wang Q, Ding S, Dong X, Xue L (2021). Red blood cell membrane-camouflaged nanoparticles loaded with AIEgen and poly(I : C) for enhanced tumoral photodynamic-immunotherapy. Natl Sci Rev.

[7] Villa CH, Anselmo AC, Mitragotri S, Muzykantov V (2016). Red blood cells: supercarriers for drugs, biologicals, and nanoparticles and inspiration for advanced delivery systems. Adv Drug Deliv Rev.

[8] Ihler G, Lantzy A, Purpura J, Glew RH (1975). Enzymatic degradation of uric acid by uricase-loaded human erythrocytes. J Clin Invest.

[9] Wang C, Wang M, Zhang Y, Jia H, Chen B (2022). Cyclic arginine-glycine-aspartic acid-modified red blood cells for drug delivery: synthesis and in vitro evaluation. J Pharm Anal.

[10] Peng H, Zhang X, Yang P, Zhao J, Zhang W, Feng N (2023). Defect self-assembly of metal-organic framework triggers ferroptosis to overcome resistance. Bioactive Mater.

[11] Zhang X, Luo M, Dastagir SR, Nixon M, Khamhoung A, Schmidt A (2021). Engineered red blood cells as an off-the-shelf allogeneic anti-tumor therapeutic. Nat Commun.

[12] Hou K, Zhang Y, Bao M, Xin C, Wei Z, Lin G (2022). A multifunctional magnetic red blood cell-mimetic micromotor for drug delivery and image-guided therapy. ACS Appl Mater Interfaces.

[13] Mesarec L, Góźdź W, Iglič A, Kralj-Iglič V, Virga EG, Kralj S (2019). Normal red blood cells’ shape stabilized by membrane’s in-plane ordering. Sci Rep.

[14] Renoux C, Faivre M, Bessaa A, Da Costa L, Joly P, Gauthier A (2019). Impact of surface-area-to-volume ratio, internal viscosity and membrane viscoelasticity on red blood cell deformability measured in isotonic condition. Sci Rep.

[15] Mohandas N, Gallagher PG (2008). Red cell membrane: past, present, and future. Blood.

[16] Svetina S (2012). Red blood cell shape and deformability in the context of the functional evolution of its membrane structure. Cell Mol Biol Lett.

[17] Li N, Chen S, Xu K, He M-T, Dong M-Q, Zhang QC (2023). Structural basis of membrane skeleton organization in red blood cells. Cell.

[18] Lux SE (2016). Anatomy of the red cell membrane skeleton: unanswered questions. Blood.

[19] Setty BNKS, Stuart MJ (2002). Role of erythrocyte phosphatidylserine in sickle red cell-endothelial adhesion. Blood.

[20] Razizadeh M, Nikfar M, Paul R, Liu Y (2020). Coarse-grained modeling of pore dynamics on the red blood cell membrane under large deformations. Biophys J.

[21] Baskurt OK, Meiselman HJ (2003). Blood rheology and hemodynamics. Semin Thromb Hemost.

[22] Virtanen JA, Somerharju KHCP (1998). Phospholipid composition of the mammalian red cell membrane can be rationalized by a superlattice model. Proc Natl Acad Sci USA.

[23] de Back DZ, Kostova EB, van Kraaij M, van den Berg TK, van Bruggen R (2014). Of macrophages and red blood cells; a complex love story. Front Physiol.

[24] Bardyn M, Rappaz B, Jaferzadeh K, Crettaz D, Tissot J-D, Moon I (2017). Red blood cells ageing markers: a multi-parametric analysis. Blood Transfus.

[25] Dzierzak E, Philipsen S (2013). Erythropoiesis: development and differentiation. Cold Spring Harb Perspect Med.

[26] Lam LKM, Murphy S, Kokkinaki D, Venosa A, Sherrill-Mix S, Casu C (2021). DNA binding to TLR9 expressed by red blood cells promotes innate immune activation and anemia. Sci Transl Med.

[27] Sahoo K, Karumuri S, Hikkaduwa Koralege RS, Flynn NH, Hartson S, Liu J (2017). Molecular and Biocompatibility characterization of red blood cell membrane targeted and cell-penetrating-peptide-modified polymeric nanoparticles. Mol Pharm.

[28] Gedde MM, Davis DK, Huestis WH (1997). Cytoplasmic pH and human erythrocyte shape. Biophys J.

[29] Krueger M, Thom F (1997). Deformability and stability of erythrocytes in high-frequency electric fields down to subzero temperatures. Biophys J.

[30] Chu X, Yu X, Greenstein J, Aydin F, Uppaladadium G, Dutt M (2017). Flow-induced shape reconfiguration, phase separation, and rupture of bio-inspired vesicles. ACS Nano.

[31] Paulitschke M, Nash GB, Anstee DJ, Tanner MJ, Gratzer WB (1995). Perturbation of red blood cell membrane rigidity by extracellular ligands. Blood.

[32] Fei Z, Fan Q, Dai H, Zhou X, Xu J, Ma Q (2021). Physiologically triggered injectable red blood cell-based gel for tumor photoablation and enhanced cancer immunotherapy. Biomaterials.

[33] Guo J, Agola JO, Serda R, Franco S, Lei Q, Wang L (2020). Biomimetic rebuilding of multifunctional red blood cells: modular design using functional components. ACS Nano.

[34] Gao C, Lin Z, Wang D, Wu Z, Xie H, He Q (2019). Red blood cell-mimicking micromotor for active photodynamic cancer therapy. ACS Appl Mater Interfaces.

[35] Dey P, Banerjee S, Mandal S, Chattopadhyay P (2019). Design and evaluation of anti-fibrosis drug engineered resealed erythrocytes for targeted delivery. Drug Deliv Transl Res.

[36] Hamidi M, Tajerzadeh H, Dehpour AR, Rouini MR, Ejtemaee-Mehr S (2001). In vitro characterization of human intact erythrocytes loaded by enalaprilat. Drug Deliv.

[37] Cheng Z, Liu S, Wu X, Raza F, Li Y, Yuan W (2020). Autologous erythrocytes delivery of berberine hydrochloride with long-acting effect for hypolipidemia treatment. Drug Deliv.

[38] Xu E, Wu X, Zhang X, Zul K, Raza F, Su J (2020). Study on the protection of dextran on erythrocytes during drug loading. Colloids Surf B Biointerfaces.

[39] Robert M, Laperrousaz B, Piedrahita D, Gautier E-F, Nemkov T, Dupuy F (2022). Multiparametric characterization of red blood cell physiology after hypotonic dialysis based drug encapsulation process. Acta Pharm Sin B.

[40] Chettab K, Matera E-L, Lafond M, Coralie D, Favin-Lévêque C, Goy C (2022). Proof of Concept: protein delivery into human erythrocytes using stable cavitation. Mol Pharm.

[41] Piergiovanni M, Casagrande G, Taverna F, Corridori I, Frigerio M, Bianchi E (2020). Shear-Induced encapsulation into red blood cells: a new microfluidic approach to drug delivery. Ann Biomed Eng.

[42] Kinosita K, Tsong TY (1978). Survival of sucrose-loaded erythrocytes in the circulation. Nature.

[43] Favretto ME, Cluitmans JCA, Bosman GJCGM, Brock R (2013). Human erythrocytes as drug carriers: loading efficiency and side effects of hypotonic dialysis, chlorpromazine treatment and fusion with liposomes. J Control Release.

[44] Ihler GM, Schnure RHGFW (1973). Enzyme loading of erythrocytes. Proc Natl Acad Sci USA.

[45] Kwon YM, Chung HS, Moon C, Yockman J, Park YJ, Gitlin SD (2009). l-Asparaginase encapsulated intact erythrocytes for treatment of acute lymphoblastic leukemia (ALL). J Control Release.

[46] Shen Y, Du K, Zou L, Zhou X, Lv R, Gao D (2019). Rapid and continuous on-chip loading of trehalose into erythrocytes. Biomed Microdevices.

[47] Hamidi M, Zarrin AH, Foroozesh M, Zarei N, Mohammadi-Samani S (2007). Preparation and in vitro evaluation of carrier erythrocytes for RES-targeted delivery of interferon-alpha 2b. Int J Pharm.

[48] Gutiérrez Millán C, Bax BE, Castañeda AZ, Marinero MLS, Lanao JM (2008). In vitro studies of amikacin-loaded human carrier erythrocytes. Transl Res.

[49] Harisa GE-dI, Ibrahim MF, Alanazi FK (2011). Characterization of human erythrocytes as potential carrier for pravastatin: an in vitro study. Int J Med Sci.

[50] Brenner JS, Mitragotri S, Muzykantov VR (2021). Red blood cell hitchhiking: a novel approach for vascular delivery of nanocarriers. Annu Rev Biomed Eng.

[51] Wang Y, Zhou C, Ding Y, Liu M, Tai Z, Jin Q (2021). Red blood cell-hitchhiking chitosan nanoparticles for prolonged blood circulation time of vitamin K(1). Int J Pharm.

[52] Li M, Xu X, Shi R, Li Y, Lin Q, Gong T (2022). Smart erythrocyte-hitchhiking insulin delivery system for prolonged automatic blood glucose control. Biomater Sci.

[53] Wang C, Ye Y, Sun W, Yu J, Wang J, Lawrence DS (2017). Red blood cells for glucose-responsive insulin delivery. Adv Mater.

[54] Feng Y, Liu Q, Li Y, Han Y, Liang M, Wang H (2021). Cell relay-delivery improves targeting and therapeutic efficacy in tumors. Bioact Mater.

[55] Zheng J, Lu C, Ding Y, Zhang J, Tan F, Liu J (2022). Red blood cell-hitchhiking mediated pulmonary delivery of ivermectin: Effects of nanoparticle properties. Int J Pharm.

[56] Zhao Z, Kim J, Suja VC, Kapate N, Gao Y, Guo J (2022). Red blood cell anchoring enables targeted transduction and re-administration of AAV-mediated gene therapy. Adv Sci.

[57] Li J, Ding Y, Cheng Q, Gao C, Wei J, Wang Z (2022). Supramolecular erythrocytes-hitchhiking drug delivery system for specific therapy of acute pneumonia. J Control Release.

[58] Ferguson LT, Hood ED, Shuvaeva T, Shuvaev VV, Basil MC, Wang Z (2022). Dual affinity to RBCs and target cells (DART) enhances both organ- and cell type-targeting of intravascular nanocarriers. ACS Nano.

[59] Ding Y, Lv B, Zheng J, Lu C, Liu J, Lei Y (2022). RBC-hitchhiking chitosan nanoparticles loading methylprednisolone for lung-targeting delivery. J Control Release.

[60] Zelepukin IV, Yaremenko AV, Shipunova VO, Babenyshev AV, Balalaeva IV, Nikitin PI (2019). Nanoparticle-based drug delivery via RBC-hitchhiking for the inhibition of lung metastases growth. Nanoscale.

[61] Ukidve A, Zhao Z, Fehnel A, Krishnan V, Pan DC, Gao Y (2020). Erythrocyte-driven immunization via biomimicry of their natural antigen-presenting function. Proc Natl Acad Sci USA.

[62] Yao H, Wang Z, Wang N, Deng Z, Liu G, Zhou J (2022). Enhancing circulation and tumor accumulation of carboplatin via an erythrocyte-anchored prodrug strategy. Angew Chem Int Ed Engl.

[63] Wang N, Deng Z, Zhu Q, Zhao J, Xie K, Shi P (2021). An erythrocyte-delivered photoactivatable oxaliplatin nanoprodrug for enhanced antitumor efficacy and immune response. Chem Sci.

[64] Gupta A, Das R, Makabenta JM, Gupta A, Zhang X, Jeon T (2021). Erythrocyte-mediated delivery of bioorthogonal nanozymes for selective targeting of bacterial infections. Mater Horiz.

[65] Zhao Z, Ukidve A, Gao Y, Kim J, Mitragotri S (2019). Erythrocyte leveraged chemotherapy (ELeCt): nanoparticle assembly on erythrocyte surface to combat lung metastasis. Sci Adv.

[66] Pan DC, Myerson JW, Brenner JS, Patel PN, Anselmo AC, Mitragotri S (2018). Nanoparticle properties modulate their attachment and effect on carrier red blood cells. Sci Rep.

[67] Chen Z, Wang W, Li Y, Wei C, Zhong P, He D (2021). Folic acid-modified erythrocyte membrane loading dual drug for targeted and chemo-photothermal synergistic cancer therapy. Mol Pharm.

[68] Liang S, Wang M, Wang J, Chen G (2021). Red-blood-cell-membrane-coated metal-drug nanoparticles for enhanced chemotherapy. ChemBioChem.

[69] Lenders V, Escudero R, Koutsoumpou X, Armengol Álvarez L, Rozenski J, Soenen SJ (2022). Modularity of RBC hitchhiking with polymeric nanoparticles: testing the limits of non-covalent adsorption. J Nanobiotechnol.

[70] Nikfar M, Razizadeh M, Paul R, Muzykantov V, Liu Y (2021). A numerical study on drug delivery via multiscale synergy of cellular hitchhiking onto red blood cells. Nanoscale.

[71] Guo Y, Wang D, Song Q, Wu T, Zhuang X, Bao Y (2015). Erythrocyte membrane-enveloped polymeric nanoparticles as nanovaccine for induction of antitumor immunity against melanoma. ACS Nano.

[72] Bao Y, Hu Q, Wang X, Feng X, He Y, Guo Y (2020). Chemo-immunotherapy with doxorubicin prodrug and erythrocyte membrane-enveloped polymer nano-vaccine enhances antitumor activity. Biomed Pharmacother.

[73] Kuo Y-C, Wu H-C, Hoang D, Bentley WE, D’Souza WD, Raghavan SR (2016). Colloidal properties of nanoerythrosomes derived from bovine red blood cells. Langmuir.

[74] Yuan J, Yin WY, Wang Y, Chen J, Zhang ZM, Tang YX (2021). Cargo-laden erythrocyte ghosts target liver mediated by macrophages. Transfus Apher Sci.

[75] Li H, Jin K, Luo M, Wang X, Zhu X, Liu X (2019). Size dependency of circulation and biodistribution of biomimetic nanoparticles: red blood cell membrane-coated nanoparticles. Cells.

[76] Jia W, Burns JM, Villantay B, Tang JC, Vankayala R, Lertsakdadet B (2020). Intravital vascular phototheranostics and real-time circulation dynamics of micro- and nanosized erythrocyte-derived carriers. ACS Appl Mater Interfaces.

[77] AlQahtani SA, Harisa GI, Badran MM, AlGhamdi KM, Kumar A, Salem-Bekhit MM (2019). Nano-erythrocyte membrane-chaperoned 5-fluorouracil liposomes as biomimetic delivery platforms to target hepatocellular carcinoma cell lines. Artif Cells Nanomed Biotechnol.

[78] Wang T, Luo Y, Lv H, Wang J, Zhang Y, Pei R (2019). Aptamer-based erythrocyte-derived mimic vesicles loaded with siRNA and doxorubicin for the targeted treatment of multidrug-resistant tumors. ACS Appl Mater Interfaces.

[79] Capossela S, Mathew V, Boos M, Bertolo A, Krupkova O, Stoyanov JV (2020). Novel fast and reliable method for nano-erythrosome production using shear force. Drug Des Devel Ther.

[80] Zhang J, Wei K, Shi J, Zhu Y, Guan M, Fu X (2021). Biomimetic nanoscale erythrocyte delivery system for enhancing chemotherapy via overcoming biological barriers. ACS Biomater Sci Eng.

[81] Della Pelle G, Delgado López A, Salord Fiol M, Kostevšek N (2021). Cyanine dyes for photo-thermal therapy: a comparison of synthetic liposomes and natural erythrocyte-based carriers. Int J Mol Sci.

[82] Li Q, Lin B, Li Y, Lu N (2021). Erythrocyte-camouflaged mesoporous titanium dioxide nanoplatform for an ultrasound-mediated sequential therapies of breast cancer. Int J Nanomed.

[83] Xuan M, Shao J, Zhao J, Li Q, Dai L, Li J (2018). Magnetic mesoporous silica nanoparticles cloaked by red blood cell membranes: applications in cancer therapy. Angew Chem Int Ed Engl.

[84] Wang P, Wang X, Luo Q, Li Y, Lin X, Fan L (2019). Fabrication of red blood cell-based multimodal theranostic probes for second near-infrared window fluorescence imaging-guided tumor surgery and photodynamic therapy. Theranostics.

[85] Lejeune AMM, Gicquaud C, Lacroix J, Poyet P, Gaudreault R (1994). Nanoerythrosome, a new derivative of erythrocyte ghost: preparation and antineoplastic potential as drug carrier for daunorubicin. Anticancer Res.

[86] Daniyal M, Jian Y, Xiao F, Sheng W, Fan J, Xiao C (2020). Development of a nanodrug-delivery system camouflaged by erythrocyte membranes for the chemo/phototherapy of cancer. Nanomedicine.

[87] Hsieh CC, Kang ST, Lin YH, Ho YJ, Wang CH, Yeh CK (2015). Biomimetic acoustically-responsive vesicles for theranostic applications. Theranostics.

[88] Wang Y, Ji X, Ruan M, Liu W, Song R, Dai J (2018). Worm-like biomimetic nanoerythrocyte carrying siRNA for melanoma gene therapy. Small.

[89] Della Pelle G, Kostevšek N (2021). Nucleic acid delivery with red-blood-cell-based carriers. Int J Mol Sci.

[90] Liu Y, Lu Y, Ning B, Su X, Yang B, Dong H (2022). Intravenous delivery of living listeria monocytogenes elicits gasdmermin-dependent tumor pyroptosis and motivates anti-tumor immune response. ACS Nano.

[91] Wu P, Jiang X, Yin S, Yang Y, Liu T, Wang K (2021). Biomimetic recombinant of red blood cell membranes for improved photothermal therapy. J Nanobiotechnol.

[92] Domingo C, Saurina J (2012). An overview of the analytical characterization of nanostructured drug delivery systems: towards green and sustainable pharmaceuticals: a review. Anal Chim Acta.

[93] Zhu K, Xu Y, Zhong R, Li W, Wang H, Wong YS (2023). Hybrid liposome-erythrocyte drug delivery system for tumor therapy with enhanced targeting and blood circulation. Regen Biomater.

[94] Wang X, Meng N, Wang S, Lu L, Wang H, Zhan C (2020). Factors influencing the immunogenicity and immunotoxicity of cyclic rgd peptide-modified nanodrug delivery systems. Mol Pharm.

[95] Zhu H, Li Y, Ming Z, Liu W (2021). Glucose oxidase-mediated tumor starvation therapy combined with photothermal therapy for colon cancer. Biomater Sci.

[96] Wu X, Zhang X, Feng W, Feng H, Ding Z, Zhao Q (2021). A targeted erythrocyte membrane-encapsulated drug-delivery system with anti-osteosarcoma and anti-osteolytic Effects. ACS Appl Mater Interfaces.

[97] Lin Y, Zhong Y, Chen Y, Li L, Chen G, Zhang J (2020). Ligand-modified erythrocyte membrane-cloaked metal-organic framework nanoparticles for targeted antitumor therapy. Mol Pharm.

[98] Sousa-Junior AA, Mendanha SA, Carrião MS, Capistrano G, Próspero AG, Soares GA (2020). Predictive model for delivery efficiency: erythrocyte membrane-camouflaged magnetofluorescent nanocarriers study. Mol Pharm.

[99] Wu X, Li Y, Raza F, Wang X, Zhang S, Rong R (2021). Red blood cell membrane-camouflaged tedizolid phosphate-loaded PLGA nanoparticles for bacterial-infection therapy. Pharmaceutics.

[100] Hao X, Li Q, Wang H, Muhammad K, Guo J, Ren X (2018). Red-blood-cell-mimetic gene delivery systems for long circulation and high transfection efficiency in ECs. J Mater Chem B.

[101] Zhang Y, Xia Q, Wu T, He Z, Li Y, Li Z (2021). A novel multi-functionalized multicellular nanodelivery system for non-small cell lung cancer photochemotherapy. J Nanobiotechnol.

[102] Zhai Z, Xu P, Yao J, Li R, Gong L, Yin Y (2020). Erythrocyte-mimicking paclitaxel nanoparticles for improving biodistributions of hydrophobic drugs to enhance antitumor efficacy. Drug Deliv.

[103] Feng S, Li H, Ren Y, Zhi C, Huang Y, Chen F (2020). RBC membrane camouflaged boron nitride nanospheres for enhanced biocompatible performance. Colloids Surf B Biointerfaces.

[104] Ren H, Liu J, Li Y, Wang H, Ge S, Yuan A (2017). Oxygen self-enriched nanoparticles functionalized with erythrocyte membranes for long circulation and enhanced phototherapy. Acta Biomater.

[105] Li L-L, Xu J-H, Qi G-B, Zhao X, Yu F, Wang H (2014). Core-shell supramolecular gelatin nanoparticles for adaptive and “on-demand” antibiotic delivery. ACS Nano.

[106] Liu B, Wang W, Fan J, Long Y, Xiao F, Daniyal M (2019). RBC membrane camouflaged prussian blue nanoparticles for gamabutolin loading and combined chemo/photothermal therapy of breast cancer. Biomaterials.

[107] Wang P, Jiang F, Chen B, Tang H, Zeng X, Cai D (2020). Bioinspired red blood cell membrane-encapsulated biomimetic nanoconstructs for synergistic and efficacious chemo-photothermal therapy. Colloids Surf B Biointerfaces.

[108] Miao Y, Yang Y, Guo L, Chen M, Zhou X, Zhao Y (2022). Cell membrane-camouflaged nanocarriers with biomimetic deformability of erythrocytes for ultralong circulation and enhanced cancer therapy. ACS Nano.

[109] Fan Z, Deng J, Li PY, Chery DR, Su Y, Zhu P (2019). A new class of biological materials: cell membrane-derived hydrogel scaffolds. Biomaterials.

[110] Li JQ, Zhao RX, Yang FM, Qi XT, Ye PK, Xie M (2022). An erythrocyte membrane-camouflaged biomimetic nanoplatform for enhanced chemo-photothermal therapy of breast cancer. J Mater Chem B.

[111] Xie M, Deng T, Li J, Shen H (2021). The camouflage of graphene oxide by red blood cell membrane with high dispersibility for cancer chemotherapy. J Colloid Interface Sci.

[112] Su J, Lu S, Wei Z, Li B, Li J, Sun J (2022). Biocompatible inorganic nanoagent for efficient synergistic tumor treatment with augmented Antitumor Immunity. Small.

[113] Malhotra S, Dumoga S, Joshi A, Mohanty S, Singh N (2021). Polymeric micelles coated with hybrid nanovesicles enhance the therapeutic potential of the reversible topoisomerase inhibitor camptothecin in a mouse model. Acta Biomater.

[114] Chen Y, Li Y, Liu J, Zhu Q, Ma J, Zhu X (2021). Erythrocyte membrane bioengineered nanoprobes via indocyanine green-directed assembly for single NIR laser-induced efficient photodynamic/photothermal theranostics. J Control Release.

[115] Shao J, Abdelghani M, Shen G, Cao S, Williams DS, van Hest JCM (2018). Erythrocyte membrane modified janus polymeric motors for thrombus therapy. ACS Nano.

[116] Yang Q, Xiao Y, Yin Y, Li G, Peng J (2019). Erythrocyte membrane-camouflaged IR780 and DTX coloading polymeric nanoparticles for imaging-guided cancer photo-chemo combination therapy. Mol Pharm.

[117] Zhu DM, Xie W, Xiao YS, Suo M, Zan MH, Liao QQ (2018). Erythrocyte membrane-coated gold nanocages for targeted photothermal and chemical cancer therapy. Nanotechnology.

[118] Mac JT, Vankayala R, Lee CH, Anvari B (2022). Erythrocyte-derived nanoparticles with Folate Functionalization for Near Infrared Pulsed laser-mediated photo-chemotherapy of tumors. Int J Mol Sci.

[119] Yang Z, Gao D, Guo X, Jin L, Zheng J, Wang Y (2020). Fighting immune cold and reprogramming immunosuppressive tumor microenvironment with red blood cell membrane-camouflaged nanobullets. ACS Nano.

[120] Sung S-Y, Su Y-L, Cheng W, Hu P-F, Chiang C-S, Chen W-T (2019). Graphene quantum dots-mediated theranostic penetrative delivery of drug and photolytics in deep tumors by targeted biomimetic nanosponges. Nano Lett.

[121] Wang L, Chen S, Pei W, Huang B, Niu C (2020). Magnetically targeted erythrocyte membrane coated nanosystem for synergistic photothermal/chemotherapy of cancer. J Mater Chem B.

[122] Long Y, Wu X, Li Z, Fan J, Hu X, Liu B, PEGylated (2020). WS(2) nanodrug system with erythrocyte membrane coating for chemo/photothermal therapy of cervical cancer. Biomater Sci.

[123] Li M, Cui X, Wei F, Li C, Han X (2022). RGD peptide modified erythrocyte membrane/porous nanoparticles loading Mir-137 for NIR-Stimulated theranostics of glioblastomas. Nanomaterials.

[124] Wang S, Yin Y, Song W, Zhang Q, Yang Z, Dong Z (2020). Red-blood-cell-membrane-enveloped magnetic nanoclusters as a biomimetic theranostic nanoplatform for bimodal imaging-guided cancer photothermal therapy. J Mater Chem B.

[125] Luo L, Zeng F, Xie J, Fan J, Xiao S, Wang Z (2020). A RBC membrane-camouflaged biomimetic nanoplatform for enhanced chemo-photothermal therapy of cervical cancer. J Mater Chem B.

[126] Liang X, Ye X, Wang C, Xing C, Miao Q, Xie Z (2019). Photothermal cancer immunotherapy by erythrocyte membrane-coated black phosphorus formulation. J Control Release.

[127] Zhang Y, Li Y, Xia Q, Li Y, Jin S, Mao Q (2022). Cell membrane-coated human hair nanoparticles for precise disease therapies. J Nanobiotechnol.

[128] Li S, Zhang L (2020). Erythrocyte membrane nano-capsules: biomimetic delivery and controlled release of photothermal-photochemical coupling agents for cancer cell therapy. Dalton Trans.

[129] Jiang Q, Liu Y, Guo R, Yao X, Sung S, Pang Z (2019). Erythrocyte-cancer hybrid membrane-camouflaged melanin nanoparticles for enhancing photothermal therapy efficacy in tumors. Biomaterials.

[130] Chen H, Deng J, Yao X, He Y, Li H, Jian Z (2021). Bone-targeted erythrocyte-cancer hybrid membrane-camouflaged nanoparticles for enhancing photothermal and hypoxia-activated chemotherapy of bone invasion by OSCC. J Nanobiotechnol.

[131] Kim MW, Lee G, Niidome T, Komohara Y, Lee R, Park YI (2020). Platelet-like gold nanostars for cancer therapy: the ability to treat cancer and evade immune reactions. Front Bioeng Biotechnol.

[132] Liu Y, Wang X, Ouyang B, Liu X, Du Y, Cai X (2018). Erythrocyte-platelet hybrid membranes coating polypyrrol nanoparticles for enhanced delivery and photothermal therapy. J Mater Chem B.

[133] Dehaini D, Wei X, Fang RH, Masson S, Angsantikul P, Luk BT (2017). Erythrocyte-platelet hybrid membrane coating for enhanced nanoparticle functionalization. Adv Mater.

[134] Wang D, Yao Y, Xiao Y, Chen X, Hu J, Yang X (2021). Ultrasound responsive erythrocyte membrane-derived hybrid nanovesicles with controlled drug release for tumor therapy. Nanoscale.

[135] Jiang L, Zhu Y, Luan P, Xu J, Ru G, Fu JG (2021). Bacteria-anchoring hybrid liposome capable of absorbing multiple toxins for antivirulence therapy of *Escherichia coli* infection. ACS Nano.

[136] Sun M, Duan Y, Ma Y, Zhang Q (2020). Cancer cell-erythrocyte hybrid membrane coated Gold nanocages for near infrared light-activated photothermal/radio/chemotherapy of breast cancer. Int J Nanomed.

[137] Xie Q, Liu Y, Long Y, Wang Z, Jiang S, Ahmed R (2021). Hybrid-cell membrane-coated nanocomplex-loaded chikusetsusaponin IVa methyl ester for a combinational therapy against breast cancer assisted by Ce6. Biomater Sci.

[138] Huo Y-Y, Song X, Zhang W-X, Zhou Z-L, Lv Q-Y, Cui H-F (2022). Thermosensitive biomimetic hybrid membrane camouflaged hollow gold nanoparticles for NIR-responsive mild-hyperthermia chemo-/photothermal combined tumor therapy. ACS Appl Bio Mater.

[139] Xiong J, Wu M, Chen J, Liu Y, Chen Y, Fan G (2021). Cancer-erythrocyte hybrid membrane-camouflaged magnetic nanoparticles with enhanced photothermal-immunotherapy for ovarian cancer. ACS Nano.

[140] Yu H, Fan J, Shehla N, Qiu Y, Lin Y, Wang Z (2022). Biomimetic Hybrid membrane-coated Xuetongsu assisted with laser irradiation for efficient rheumatoid arthritis therapy. ACS Nano.

[141] Xu L, Liang Y, Xu X, Xia J, Wen C, Zhang P (2021). Blood cell-derived extracellular vesicles: diagnostic biomarkers and smart delivery systems. Bioengineered.

[142] Sorkin R, Huisjes R, Bošković F, Vorselen D, Pignatelli S, Ofir-Birin Y (2018). Nanomechanics of extracellular vesicles reveals vesiculation pathways. Small.

[143] Wu SH, Hsieh CC, Hsu SC, Yao M, Hsiao JK, Wang SW (2021). RBC-derived vesicles as a systemic delivery system of doxorubicin for lysosomal-mitochondrial axis-improved cancer therapy. J Adv Res.

[144] Hanley TM, Vankayala R, Mac JT, Lo DD, Anvari B (2020). Acute immune response of micro- and nanosized erythrocyte-derived optical particles in healthy mice. Mol Pharm.

[145] Fan X, Xu H, Zhao F, Song J, Jin Y, Zhang C (2020). Lipid-mimicking peptide decorates erythrocyte membrane for active delivery to engrafted MDA-MB-231 breast tumour. Eur J Pharm Biopharm.

[146] Borgheti-Cardoso LN, Kooijmans SAA, Chamorro LG, Biosca A, Lantero E, Ramírez M (2020). Extracellular vesicles derived from Plasmodium-infected and non-infected red blood cells as targeted drug delivery vehicles. Int J Pharm.

[147] Zhang Y, Wang Y, Xin Q, Li M, Yu P, Luo J (2022). Zwitterionic choline phosphate conjugated folate-poly (ethylene glycol): a general decoration of erythrocyte membrane-coated nanoparticles for enhanced tumor-targeting drug delivery. J Mater Chem B.

[148] Liu Y, Bhattarai P, Dai Z, Chen X (2019). Photothermal therapy and photoacoustic imaging via nanotheranostics in fighting cancer. Chem Soc Rev.

[149] Bao Y, Chen J, Qiu H, Zhang C, Huang P, Mao Z (2021). Erythrocyte membrane-camouflaged PCN-224 nanocarriers integrated with platinum nanoparticles and glucose oxidase for enhanced tumor sonodynamic therapy and synergistic starvation therapy. ACS Appl Mater Interfaces.

[150] Peng J, Yang Q, Li W, Tan L, Xiao Y, Chen L (2017). Erythrocyte-membrane-coated prussian blue/manganese dioxide nanoparticles as H2O2-responsive oxygen generators to enhance cancer chemotherapy/photothermal therapy. ACS Appl Mater Interfaces.

[151] Shao J, Feng L, Zhao Q, Chen C, Li J, Ma Q (2022). Erythrocyte-mimicking subcutaneous platform with a laser-controlled treatment against diabetes. J Control Release.

[152] Vankayala R, Mac JT, Burns JM, Dunn E, Carroll S, Bahena EM (2019). Biodistribution and toxicological evaluation of micron- and nano-sized erythrocyte-derived optical particles in healthy Swiss Webster mice. Biomater Sci.

[153] Wu Z, Li T, Li J, Gao W, Xu T, Christianson C (2014). Turning erythrocytes into functional micromotors. ACS Nano.

[154] Antonelli A, Pacifico S, Sfara C, Tamma M, Magnani M (2018). Ferucarbotran-loaded red blood cells as long circulating MRI contrast agents: first in vivo results in mice. Nanomedicine.

[155] Chang M, Hsiao J-K, Yao M, Chien L-Y, Hsu S-C, Ko B-S (2010). Homologous RBC-derived vesicles as ultrasmall carriers of iron oxide for magnetic resonance imaging of stem cells. Nanotechnology.

[156] Marvin CM, Ding S, White RE, Orlova N, Wang Q, Zywot EM (2019). On command drug delivery via cell-conveyed phototherapeutics. Small.

[157] Zhu YX, Jia HR, Guo Y, Liu X, Zhou N, Liu P (2021). Repurposing erythrocytes as a “photoactivatable bomb”: a general strategy for site-specific drug release in blood vessels. Small.

[158] Wu Z, Esteban-Fernández de Ávila B, Martín A, Christianson C, Gao W, Thamphiwatana SK (2015). RBC micromotors carrying multiple cargos towards potential theranostic applications. Nanoscale.

[159] Wang C, Sun X, Cheng L, Yin S, Yang G, Li Y (2014). Multifunctional theranostic red blood cells for magnetic-field-enhanced in vivo combination therapy of cancer. Adv Mater.

[160] Rao L, Meng Q-F, Bu L-L, Cai B, Huang Q, Sun Z-J (2017). Erythrocyte membrane-coated upconversion nanoparticles with minimal protein adsorption for enhanced tumor imaging. ACS Appl Mater Interfaces.

[161] Li M, Fang H, Liu Q, Gai Y, Yuan L, Wang S (2020). Red blood cell membrane-coated upconversion nanoparticles for pretargeted multimodality imaging of triple-negative breast cancer. Biomater Sci.

[162] Han S, Wang W, Wang S, Wang S, Ju R, Pan Z (2019). Multifunctional biomimetic nanoparticles loading baicalin for polarizing tumor-associated macrophages. Nanoscale.

[163] Geng Z, Chen F, Wang X, Wang L, Pang Y, Liu J (2021). Combining anti-PD-1 antibodies with mn(2+)-drug coordinated multifunctional nanoparticles for enhanced cancer therapy. Biomaterials.

[164] Han X, Shen S, Fan Q, Chen G, Archibong E, Dotti G (2019). Red blood cell-derived nanoerythrosome for antigen delivery with enhanced cancer immunotherapy. Sci Adv.

[165] Mambrini G, Mandolini M, Rossi L, Pierigè F, Capogrossi G, Salvati P (2017). Ex vivo encapsulation of dexamethasone sodium phosphate into human autologous erythrocytes using fully automated biomedical equipment. Int J Pharm.

[166] Zhang X, Qiu M, Guo P, Lian Y, Xu E, Su J (2018). Autologous red blood cell delivery of betamethasone phosphate sodium for long anti-inflammation. Pharmaceutics.

[167] Wang Z, Cheng L, Sun Y, Wei X, Cai B, Liao L (2021). Enhanced isolation of fetal nucleated red blood cells by enythrocyte-leukocyte hybrid membrane-coated magnetic nanoparticles for noninvasive pregnant diagnostics. Anal Chem.

[168] Liu X, Zhang L, Jiang W, Yang Z, Gan Z, Yu C (2020). In vitro and in vivo evaluation of liposomes modified with polypeptides and red cell membrane as a novel drug delivery system for myocardium targeting. Drug Deliv.

[169] Viallat A, Abkarian M (2014). Red blood cell: from its mechanics to its motion in shear flow. Int J Lab Hematol.

[170] Bosman GJ, Cluitmans JC, Groenen YA, Werre JM, Willekens FL, Novotný VM (2011). Susceptibility to hyperosmotic stress-induced phosphatidylserine exposure increases during red blood cell storage. Transfusion.

[171] Lapillonne H, Kobari L, Mazurier C, Tropel P, Giarratana M-C, Zanella-Cleon I (2010). Red blood cell generation from human induced pluripotent stem cells: perspectives for transfusion medicine. Haematologica.

[172] Cho YK, Kim H-K, Kwon SS, Jeon S-H, Cheong J-W, Nam KT (2023). In vitro erythrocyte production using human-induced pluripotent stem cells: determining the best hematopoietic stem cell sources. Stem Cell Res Ther.

[173] Wang Y, Gao S, Zhu K, Ren L, Yuan X (2023). Integration of Trehalose lipids with dissociative trehalose enables cryopreservation of human RBCs. ACS Biomater Sci Eng.

[174] Chakkumpulakkal Puthan Veettil T, Alves D, Vongsvivut J, Sparrow RL, Wood BR, Garnier G (2023). Characterization of freeze-dried oxidized human red blood cells for pre-transfusion testing by synchrotron FTIR microspectroscopy live-cell analysis. Analyst.

[175] Stoll C, Stadnick H, Kollas O, Holovati JL, Glasmacher B, Acker JP (2011). Liposomes alter thermal phase behavior and composition of red blood cell membranes. Biochim Biophys Acta.

